# UHPLC-MS Metabolome Fingerprinting: The Isolation of Main Compounds and Antioxidant Activity of the Andean Species *Tetraglochin ameghinoi* (Speg.) Speg.

**DOI:** 10.3390/molecules23040793

**Published:** 2018-03-29

**Authors:** Lorena Luna, Mario J. Simirgiotis, Beatriz Lima, Jorge Bórquez, Gabriela E. Feresin, Alejandro Tapia

**Affiliations:** 1 Instituto de Biotecnología-Instituto de Ciencias Básicas, Universidad Nacional de San Juan, Av. Li bertador General San Martín 1109 (O), San Juan CP5400, Argentina; lluna@unsj.edu.ar (L.L.); blima@unsj.edu.ar (B.L.); gferesin@unsj.edu.ar (G.E.F.); 2 Instituto de Farmacia, Facultad de Ciencias, Universidad Austral de Chile, Campus Isla Teja, Valdivia 5090000, Chile; 3 Center for Interdisciplinary Studies on the Nervous System (CISNe), Universidad Austral de Chile, Valdivia 5090000, Chile; 4 CONICET (Consejo Nacional de Ciencia y Tecnología), CABA, Buenos Aires C1405DJR, Argentina; 5 Laboratorio de Productos Naturales Depto. de Química, Facultad de Ciencias, Universidad de Antofagasta, Av. Coloso S-N, Antofagasta 1240000, Chile; jorge.borquez@uantof.cl

**Keywords:** acetophenones, antioxidant, phenolic, UHPLC Orbitrap (Q-OT), triterpenes

## Abstract

The seriated extracts of petroleum ether (PE-E), dichloromethane (DCM-E) and methanol extracts (MeOH-E) from the aerial parts of the native South American plant *Tetraglochin ameghinoi* (Rosaceae), were evaluated regarding their antioxidant and antibacterial activities. The antioxidant properties were evaluated by free radical scavenging methods (DPPH and TEAC), ferric-reducing antioxidant power (FRAP) and lipoperoxidation in erythrocytes (LP), while the antibacterial activity was performed against Gram-positive and Gram-negative bacteria according to the Clinical and Laboratory Standards Institute (CLSI) guidelines. The chemical and biological analyses of this plant are very important since this bush is currently used in traditional medicine as a cholagogue and digestive. The polar MeOH-E showed the highest antioxidant activities (17.70 µg/mL in the DPPH assay, 381.43 ± 22.38 mM TE/g extract in the FRAP assay, 387.76 ± 91.93 mg TE/g extract in the TEAC assay and 93.23 + 6.77% in the LP assay) and it was selected for chromatographic isolation of its components. These components were found to be four acetophenones, including the new phloracetophenone glucoside: 4′,6′,-dihydroxy-2′-*O*-(6″-acetyl)-*β*-d-glucopyranosylacetophenone or IUPAC name: (6-(2-acetyl-3,5-dihydroxyphenoxy)-3,4,5-trihydroxytetrahydro-2H-pyran-2-yl)methyl acetate, whose structure was elucidated by NMR and MS methods. In addition, twenty-six compounds, including five of these acetophenone derivatives, two sugars, six flavonoids, eleven phenolic acids and two triterpenes, were identified based on UHPLC-OT-MS and PDA analysis on the MeOH-E. The results support the medicinal use of the plant.

## 1. Introduction

The numerous medicinal plants growing in the Andes of Argentina are collected annually by the inhabitants of these regions and are sold in popular local markets for their therapeutic properties. Indeed, a significant percentage of these species belonging to the genera *Azorella*, *Baccharis*, *Tetraglochin* and *Senecio* are used in local traditional medicine, which mostly aims to treat digestive and hepatic disorders, fevers, coughs and colds.

The *Tetraglochin* genus, which belongs to the Rosaceae family, are distributed along the Andes from Peru to southern Argentina, including central Chile. This genus comprises of the following eight species: *Tetraglochin acanthocarpum* (Speg.) Speg.; *T. alatum* (Gillies ex Hook. & Arn.) Kuntze; *T. ameghinoi* (Speg.) Speg.; *T. caespitosum* Phil.; *T. cristatum* (Britton) Rothm.; *T. inerme* (I. M. Johnst.) Rothm.; *T. macrophyllus* (Phil.) Niederl.; and *T. paucijugata* I. M. Johnst [1]. Recently, *T. andina* Ciald has been proposed by Acosta et al. [2] as a new species. The aerial parts of *T. ameghinoi* are employed as infusions and/or decoctions in Andean traditional medicine as cholagogues and digestives to treat hepatic disorders or bacterial infections and food-borne illnesses associated with enterobacteria. In addition, due to their medicinal properties, they are commercially exploited [3]. No previous reports about phytochemical studies and bioactive properties have been reported.

The use of HPLC or UHPLC coupled to hybrid state-of-the-art mass spectrometers, such as quadrupole orbitrap (Q-OT), is becoming a key tool for the rapid analysis of phenolics compounds in plant samples and other biological matrices. A considerable number of Andean species, mainly from Chile, have been recently analyzed using this technology [4,5,6,7,8,9].

The main goals and novelty of this work are the metabolome profiling using a hybrid high resolution mass spectrometer with an orbital trap (Q-Exactive Focus), the isolation of a main new acetophenone compound and the investigation of the antioxidant and antibacterial effects of extracts from the native medicinal plant *T. ameghinoi*, which have not yet been reported.

## 2. Results and Discussion

Seriated extracts (PE-E, DCM-E, EtOAc-E and MeOH-E extracts) prepared from aerial parts of *T. ameghinoi* were assessed in vitro for antioxidant and antimicrobial properties in addition to total content of phenolics and flavonoids. The following sections contain an explanation of the assays. 

### 2.1. Total Phenolic and Flavonoids Content

The MeOH extract (Table 1) showed a high content of phenolic compounds (107 mg GAE/g extract) and among them, 20% corresponded to flavonoids (19 mg QE/g extract). Regarding the EtOAc-E, it is important to note the amount of phenolics (45 mg GAE/g extract) and the lack of any detected flavonoids.

During the last few decades, more than 9000 flavonoids have been identified in plants. The flavonoids have been recognized as chemical compounds with a preponderant role in the human diet, highlighted by their powerful antioxidant activities, which are vital for maintaining good human health and preventing associated diseases from a direct or indirect role from oxidative stress. The versatile health benefits of flavonoids include anti-inflammatory, antioxidant, hepatoprotective, antiproliferative and anticancer activity, free radical scavenging capacity, antihypertensive effects, coronary heart disease prevention and anti-human immunodeficiency virus functions [10,11]. Different flavonoids have been investigated for their potential antiviral activities and several of them exhibited significant antiviral properties in in vitro and even in vivo studies. [12].

The chemical nature of flavonoids depends on their structural class, degree of hydroxylation, glycosylation of hydroxyl groups or of the flavonoids nucleus, other substitutions and conjugations and the degree of polymerization [13].

### 2.2. Antioxidant Activity

Reactive oxygen species (ROS) are derived from many sources, including mitochondria, xanthine oxidase, uncoupled nitric oxide synthases and NADPH oxidase [14,15].

Oxidative stress mainly caused by reactive oxygen species (ROS) damage normal organs, leading to a gradual loss of vital physiological function. Liver diseases are considered as serious problems, which can be caused by toxic chemicals, drugs, viral infiltration through ingestion or infection and the metabolic or drug/chemical-induced liver damage. Therefore, antioxidants can be used to protect the liver, which act on the inhibition of free radical generation and can decrease the oxidative damage by directly inhibiting the activity or expression of free radical generating enzymes or enhancing the activity or expression of intracellular antioxidant enzymes [16,17,18].

Polar extracts obtained from *T. ameghinoi* showed strong free radical scavenging activity in the DPPH, FRAP, ABTS and lipid peroxidation in erythrocytes assays (Table 1). The non-polar extracts PE-E and DCM-E did not have any activity in the antioxidant assays, (Table 1), which is possibly due to the presence of waxes and fats and lack of no phenolic compounds. The DPPH assay is widely used for quickly assessing the ability of polyphenols to transfer labile H atoms to radicals, which is a likely mechanism of antioxidant protection [19]. MeOH and ethyl acetate extracts from *T. ameghinoi* showed high scavenging activities in the DPPH assay with IC_50_ values of 17.0 and 45.0 µg/mL, respectively. This could be related the presence of hydrogen donating compounds, which are more likely to be present in polar extracts. The highest antioxidant capacity detected is consistent with the highest content of total phenolics detected in both extracts. Moreover, a positive significant Pearson’s correlation (*R*^2^ = 0.89 at *p* < 0.01) was found between the total phenolics content and the DPPH activity, which suggests that the phenolic compounds could be responsible for the antioxidant capacity of *T. ameghinoi* MeOH extract.

Regarding the FRAP assay, the MeOH-E and EtOAc-E showed stronger reducing power with values of 381 and 288 mM TE/g of the extract, respectively. Additionally, both extracts were the most active in the TEAC assay (387 and 154 mg TE/g extract, respectively).

Furthermore, as a cell-based model, lipid peroxidation in human erythrocytes was studied to evaluate the biological relevance of the antioxidant activities of *T. ameghinoi* polar extracts. The highest activity was found in the MeOH-E. The results showed that the MeOH-E prevented the hemolysis caused by the rupture of cell membranes induced by lipid peroxidation (>95%, at 100 µg/mL). 

Flavonoids and phenolics of higher plants are known to be excellent antioxidants in vitro and numerous studies have suggested that dietary intake of plant polyphenol antioxidants may have positive effects in oxidative-stress related pathologies. The strong lipid peroxidation inhibition by *T. ameghinoi* polar extracts may be related to the presence of phenolic compounds [20,21,22]. 

### 2.3. Antibacterial Activity 

The *T. ameghinoi* extracts were assayed and found to be antibacterial against the pathogenic bacteria Gram-negative strains (ATCC and clinical isolates of *E. coli*), and Gram-positive methicillin sensitive (MSSA) and methicillin resistant (MRSA) *Staphylococcus aureus* strains, such as *S. aureus* coagulase negative-502 and *Streptococcus pyogenes*-1. The DCM-E and MeOH-E showed very low activity against Gram-positive bacteria (MIC = 750 µg/mL), excluding the *Streptococcus pyogenes*-1 strain (Table 1), and null activity against the rest of the bacteria in the panel (MIC values > 1000 µg/mL). Regarding the PE-E, it was not active against any of the bacteria tested (MIC values > 1000 µg/mL, data not shown). Food-borne illnesses associated with Gram-positive bacteria, including the *S. aureus* enterotoxigenic strains, are a major public health issue worldwide [23].

The extracts did not exhibit relevant antimicrobial activities. The antimicrobial activity is considered very interesting in the case of MICs < 100 μg mL^−1^ for extracts and 10 μg mL^−1^ for isolated compounds [24].

### 2.4. UHPLC-OT Analysis of MeOH-E 

**Figure 1:**
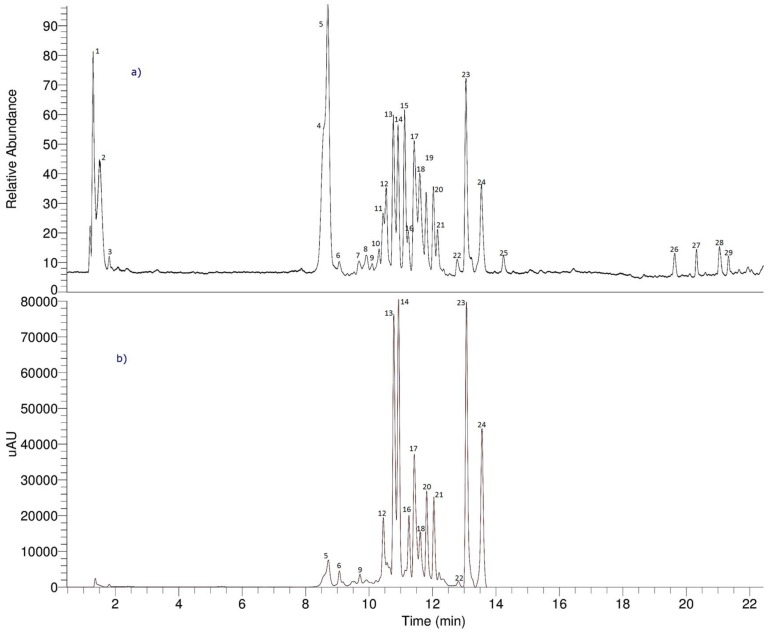
The HPLC-MS Fingerprints of MeOH-E of *T. ameghinoi*: (**a**) The total Ion Current (TIC) chromatogram and (**b**) the UV-vis chromatogram at 280 nm.

**Figure 2:**
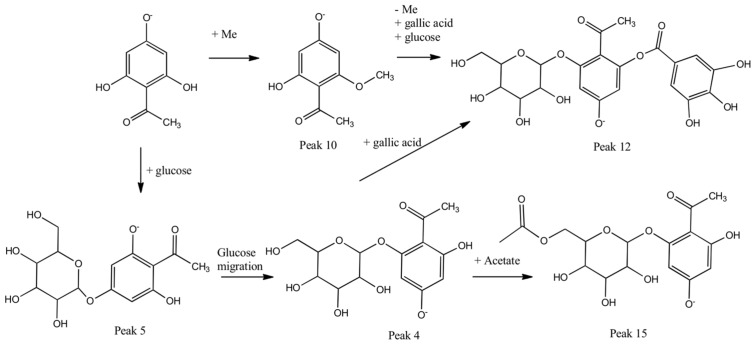
The proposed biosynthetic relationships between the acetophenone compounds detected in *T. ameghinoi*.

Since the methanol (MeOH-E) extract showed the highest biological activity, this extract was selected for detailed chemical analysis. Twenty-nine compounds were detected and twenty-six compounds were identified based on the UHPLC OT-MS and PDA analysis on MeOH-E (Figure 1 and Table 2). Several compounds were acetophenone derivatives (peaks 4, 5, 10, 12 and 15; Figure 2), two were sugars (peaks 1 and 2), several were flavonoids (peaks 16, 22–25), others were phenolic acids (peaks 6, 7, 9, 11, 13, 14, 17–20 and 28) and two were triterpenes (peaks 21 and 27). Figure 2 shows some of the biosynthetic relationships between the acetophenone compounds detected, while Figure S1 (Supplementary material) shows the full HR-MS spectra and structures of the compounds. The metabolomics identification is explained below in detail.

#### 2.4.1. Sugars

Peaks 1 and 2 with a [M − H]^−^ ion at around a *m*/*z* of 377.08365 were identified as dihexoside chloride adducts [25].

#### 2.4.2. Acetophenones

**Figure 3:**
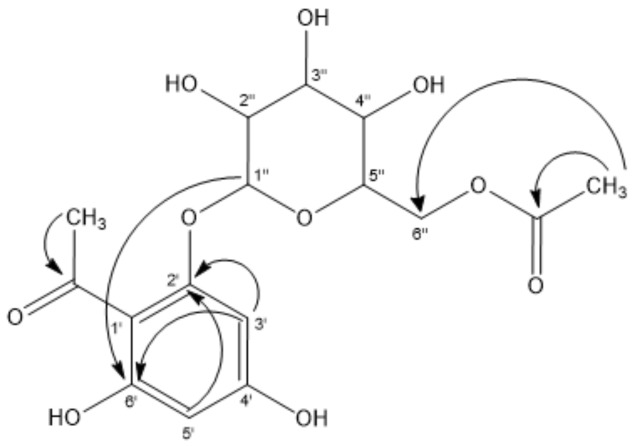
The key correlations in the corresponding HMBC spectrum of 4′,6′dihydroxy-2′-*O*-(6′′-acetyl)-*β*-d-glucopyranosylacetophenone.

Peaks 4 and 5 were the major isomer compounds showing UV maximums at *m*/*z* of 236–285 nm, with [M − H]^−^ ions at around a *m*/*z* of 329.08618 and diagnostic 2,4,6-tryhydroxyacetophenone fragments at around *m*/*z* of 167.0363 (C_8_H_7_O_4_^−^). These were identified as 4,6-dihydroxy-2′-*O*-*β*-d-glucopyranosyl acetophenone and 2,6-dihydroxy-4′-*O*-*β*-d-glucopyranosyl-acetophenone (C_14_H_17_O_9_^−^) [26], respectively, and confirmed by spiking experiments with the isolated compounds. In addition, peak 10 was identified as 2′,6′-dihydroxy-4′-*O*-methoxy-acetophenone (C_9_H_9_O_4_^−^). Section 3.3 contains details on the extraction and isolation of peaks 4, 5 and 10. Similarly, peak 12 with an anion at a *m*/*z* of 481.09613 was identified as a gallic acid glucoside derivative linked to the acetophenone fragment of 4-hydroxy-2′-*O*-*β*-d-glucopyranosyl-6-*O*-galloyl acetophenone (C_21_H_21_O_13_^−^, Figure S1). Finally, peak 15 with a pseudomolecular ion at a *m*/*z* of 371.09653 also showing a diagnostic acetophenone fragment at a *m*/*z* of 167.03355 (C_8_H_7_O_4_^−^) was identified as the acetyl derivative of the major compound 4′,6′-dihydroxy-2′-*O*-(6′′acetyl)-*β*-d-glucopyranosylacetophenone (C1_6_H_19_O_10_^−^) (Table 2; Figure S1j, Supplementary material). This new compound was obtained as white needles, before being identified based on NMR spectroscopic data, micro-melting point, UV, IR and MS. Section 3.3 contains details on the extraction and isolation. The spectroscopic data are in partly consistent with those previously published for the compound 4′,6′-dihydroxy-2′-*O*-*β*-d-glucopyranosyl-acetophenone (peak 4, Table 2, Figure S1a, Supplementary material) [27]. The signal at δ of 203.43 in the ^13^CNMR spectrum is associated with the presence of a keto function in the molecule (quaternary carbon). The ^1^HNMR spectrum revealed the presence of two aromatic protons showing a *meta* coupling δ of 5.99 (d, J = 2.4 Hz, 1H, H-5′); and δ of 6.14 (d, J = 2.4 Hz, 1H, H-3′). A singlet at δ of 2.70 accounted for a methyl group attached to an aromatic keto group, which are supported by correlations in the corresponding HMBC and HSQC experiments (see Figure 3 and Supplementary information). Furthermore, the ^1^HNMR spectrum supports the presence of a glucose moiety as follows: a signal at δ of 5.01 (d, J = 8 Hz; H-1′′) that could be assigned to the anomeric proton. In addition, the signals at δ of 4.29 (dd, J = 12.8 Hz, 1H), 4.42 (dd, J = 12. 2 Hz, 1H) and 3.37–3.69 (m, 4H) account for six protons belonging to the glucose moiety. 

The ^13^CNMR spectrum shows four non-equivalent signals corresponding to the carbons C-2′, C-6′, C-3′, and C-5′ at δ of 166.2, 161.6, 94.3 and 96.8, respectively.

The spectroscopic analysis data supports the following phloracetophenone glucoside derivatives. The first is 4′,6′-dihydroxy-2′-*O*-(6′′-acetyl)-*β*-d-glucopyranosylacetophenone with white crystals, m.p. of 209.9–211.9 °C and ^1^HNMR (CDC13) of 2.0 (s, OAc), 2.70 (s, 3H, CH_3_); 3.37–3.69 (m, 4H), 4.29 (dd, J = 12.8 Hz, 1H); 4.42 (dd, J = 12.2 Hz, 1H); 5.01 (d, J = 8 Hz, H-1′′); 5.99 (d, J = 2.4 Hz, 1H, H-5′); and 6.14 (d, J = 2.4 Hz, 1H, H-3′). The 13C NMR (CDC13) is 19.3 (OAc); 32.0 (CH_3_), 63.2 (C-6′′, CH_2_), 70.1 (C-4′′), 73.9 (C-2′′), 76.9 and 78.6 (C-3′′ and C-5′′), 96.8 (C-5′), 100.53 (C-1′′), 94.3 (C-3′), 105.5 (C-l′), 161.6 (C-6′), 166.2 (C-2′), 167.7 (C-4′), 171 (CO; OAc), and 203.4 (CO). The main phloracetophenone glucoside derivatives isolated from *T. ameghinoi* did not exhibit relevant free radical scavenging activity in terms of the DPPH, FRAP, ABTS and lipid peroxidation in erythrocytes assays (data not shown).

The choleretic activity in vivo of 4′,6′-dihydroxy-2′-*O*-(*β*-d-glucopyranosyl) acetophenone, isolated from the ethyl acetate extract of *Curcuma comosa* rhizomes, has been reported [27]. Furthermore, the aglycone of this compound stands out for its powerful choleretic activity showing both high bile flow rate and high bile salt output, which leads to lower plasma cholesterol levels [28].

This can be explained by the increase in the activation of the hepatic 7 α-hydroxylase enzyme, which suggests that the phloracetophenone exerts a “forced” effect on the liver cholesterol, favoring its conversion into bile acids for its subsequent secretion [29]. The major phenolic compounds of *T. ameghinoi*, including phloracetophenone derivatives possessing choleretic activities, has medicinal uses in traditional Andean medicine to treat liver problems and its use as a cholagogue. 

#### 2.4.3. Phenolic Acids and Derivatives

Peaks 6 and 9 were identified as gallic acid derivatives (λ around 285 nm). Peak 6 with a [M − H]^−^ ion at *m*/*z* of 453.10156 was identified as a gallic acid glucoside derivative with a molecular formula of C_20_H_21_O_12_^−^ [30], while peak 9 with a [M − H]^−^ ion at *m*/*z* of 451.12234 was identified as a dimethylgallate glucoside derivative with a molecular formula of C_21_H_23_O_11_^−^ (Figure S1, Supplementary material). Several compounds were also identified as ellagic acid derivatives (λ max around 253–367 nm). Thus, peak 7 with an anion at *m*/*z* of 463.04950 was identified as ellagic acid glucoside (C_20_H_15_O_13_^−^) [31], peak 13 was identified as its aglycon ellagic acid (C_14_H_5_O_8_^−^), while peak 14 with a ion at *m*/*z* of 477.06494 was identified as its methyl derivative of ellagic acid glucoside methyl ether (C_21_H_17_O_13_^−^). Furthermore, peaks 19 and 20 were identified as methyl ether isomers of ellagic acid (C_15_H_7_O_8_^−^) [32]. Peaks 17 and 18 with pseudomolecular ions around *m*/*z* of 394.96948 and MS2 fragments at *m*/*z* of 315.01288 (ellagic acid methyl ether) and *m*/*z* of 299.98950 (ellagic acid) were identified as isomers of methyl-ellagic acid-*O*-phosphate (C_15_H_8_O_11_P^−^). Peak 28 was identified as gingerol [33].

The oxidative stress that results from an overproduction and accumulation of free radicals is the leading cause of several degenerative diseases, such as cancer, atherosclerosis, ageing, cardiovascular and inflammatory diseases. Polyphenols form an important class of naturally occurring antioxidants, having innumerable biological activities such as anticancer, antifungal, antibacterial, antiviral, anti-ulcer and anti-cholesterol. Among the various polyphenols, gallic acid has emerged as a strong antioxidant and an efficient apoptosis inducing agent, which is a naturally occurring low molecular weight triphenolic compound [34]. 

The liver performs a vital role in the metabolism, secretion, storage and detoxification of endogenous and exogenous substances. Oxidative stress and free radicals enhance the severity of hepatic damage, which can be overcome by antioxidants. Plant extracts can be the best source of such antioxidants and mediate hepatoprotective activities.

The hepatoprotective activity of ellagic acid in comparison to silymarin using paracetamol-induced acute liver damage supports the use of this active phytochemical against toxic liver injury, which may act by preventing the lipid peroxidation and augmenting the antioxidant defense system or regeneration of hepatocytes [35].

#### 2.4.4. Flavonols

Peak 11 with a [M − H]^−^ ion at *m*/*z* of 615.09518 yielding a MS ion at *m*/*z* of 463.08565 (Quercetin glucoside) was identified as quercetin-3-*O*-(6′-*O*-galloyl)-glucose [5]. Peak 16 with a pseudomolecular ion at *m*/*z* of 433.07559 was identified as guaijaverin or quercetin-3-*O*-arabinoside (C_21_H_21_O_10_^−^) and peak 24 as the aglicone quercetin (C_15_H_9_O_7_^−^). Peak 22 with a [M − H]^−^ ion at *m*/*z* of 475.12210 was identified as the 3-*O*-rhamnoside of quercetin 3′,7-*O*-dimethyl ether, which is rhamnazin 3-*O*-rhamnoside (C_23_H_23_O_11_^−^) [36]. Peak 23 with a [M − H]^−^ ion at *m*/*z* of 593.12695 was identified as kaempferol 3-*O*-rutinoside (C_27_H_29_O_15_-) [4], while peak 25 (pseudomolecular ion at *m*/*z* of 507.11154) was identified as syringetin-3-*O*-glucoside (C_23_H_23_O_13_^−^) [7]. Quercetin galloyl glucosides suppress the oxidative metabolism in polymorphonuclear neutrophils at a comparable level to that of quercetin, although the latter was much stronger as an inhibitor of lipid peroxidation. The *ortho* arrangement of the two hydroxyls groups (free catechol grouping) in the B ring of the flavonoids, quercetin galloyl glucoside and quercetin, support their similar antioxidative properties. The activity-lowering effect of glucosidation at C-3 of the aglycone is cancelled out by the presence of the galloyl group, which is known for its antioxidative properties [37].

Some epidemiological studies have found a positive association between the consumption of foods containing kaempferol and a reduced risk of developing several disorders, such as cancer and cardiovascular diseases. Numerous preclinical studies have shown that kaempferol and their glycosides have a wide range of pharmacological activities, including antioxidant, anti-inflammatory, antimicrobial, anticancer, cardioprotective, neuroprotective, antidiabetic, anti-osteoporotic, estrogenic/antiestrogenic, anxiolytic, analgesic and anti-allergic activities [38].

Recently, the hepatoprotective effect of kaempferol-3-*O*-rutinoside (K-3-R) and kaempferol 3-*O*-glucoside (K-3-G) on the CCl_4_-induced oxidative liver injury in mice has been reported [39]. Additionally, mice treated with K-3-R and K-3-G had significantly restored glutathione (GSH) levels and showed normal catalase (CAT) and superoxide dismutase (SOD) activities compared to CCl_4_-treated mice. K-3-R and K-3-G also mitigated the CCl_4_-induced liver histological alteration, which was indicated by histopathological evaluation [39].

#### 2.4.5. Triterpenes

Pentacyclic triterpenoids, which are widely distributed in the plant kingdom, have been extensively reported to possess protective effects against drug-induced organ toxicities, including those of chemotherapeutic agents [40]. Peaks 21 and 27 with [M − H]^−^ ions at *m*/*z* of 517.31421 and *m*/*z* of 503.33493 were identified as the triterpenoid madecassic acid (C_30_H_47_O_6_^−^), which is a component of the crude drug *Centella asiatica* [41], and bartogenic acid (C_30_H_45_O_7_^−^), which is an alpha-amylase inhibitor [42], respectively.

The anti-inflammatory effects of madecassic acid have been reported, which is thought to occur via the suppression of the NF-κB pathway in LPS-induced RAW 264.7 macrophage cells [43]. The results suggest that the anti-inflammatory properties of madecassic acid are caused by iNOS, COX-2, TNF-alpha, IL-1beta and IL-6 inhibition via the downregulation of NF-κB activation in RAW 264.7 macrophage cells.

## 3. Materials and Methods 

### 3.1. General Experimental Procedures

Ultra-pure water (<5 µg/L TOC) was obtained from a water purification system Arium 126 61316-RO and an Arium 611 UV unit (Sartorius, Germany). Methanol (HPLC grade) and formic acid (puriss. p.a. for mass spectrometry) were obtained from J. T. Baker (México, México) and Fluka (Steinheim, Germany), respectively. Chloroform was obtained from Fisher, USA. The acetic acid, HCl (37%) and sulfuric acid was purchased from Merck Química Argentina (Buenos Aires, Argentina). Silica gel F254 plates (Merck), p-anisaldehyde (Aldrich Chemical Co., St Louis, MO, USA), Silica gel Kieselgel 60 (Merck) and Sephadex LH-20 (Pharmacia Inc., Bridgewater, NJ, USA) were used. The column chromatography was conducted in silica gel (Merck Química Argentina (Buenos Aires, Argentina)). Commercial Folin-Ciocalteu (FC) reagent, 2,2-Diphenyl-1-picrylhydrazyl (DPPH), ferric chloride hexahydrate, 2,4,6-tris(2-pyridyl)-s-triazine, trolox, quercetin, rutin and gallic acid (GA) were purchased from Sigma-Aldrich. Cefotaxime was from Argentia^®^ (Bristol-Myers Squibb, Buenos Aires, Argentina), Müeller–Hinton broth (Laboratorio Britania, Buenos Aires, Argentina) and DMSO were used for antibacterial testing (Merck, Darmstadt, Germany).

For the structural identification of the compounds, ^1^H-NMR spectra were recorded at 400 MHz and ^13^CNMR were obtained at 125 MHz on a Bruker spectrometer (δ scale). The TMS was employed as the internal standard. The two-dimensional experiments (COSY, HSQC, HMBC and ROESY) were applied using standard sequences. The ESI-HRMSs spectra were recorded on an orbitrap (Q-OT) mass spectrometer. The melting points were determined on a Kofler hot stage apparatus (Electrothermal 9100) and were uncorrected. The IR spectra were recorded on a Nicolet Nexus FT-IR instrument. The identification and quantification of phenolic compounds was done by a UHPLC-Q-OT-HESI-MS/MS. A Thermo Scientific Dionex Ultimate 3000 UHPLC system controlled by the Chromeleon 7.2 Software (Thermo Fisher Scientific, Waltham, MA, USA), which was hyphenated with a Thermo high resolution Q Exactive focus mass spectrometer (Thermo, Bremen, Germany). Nitrogen (purity > 99.999%) obtained from a Zefiro nitrogen generator (Clantecnologica, Sevilla, España) was employed as both the collision and damping gas. All calibration and equipment parameters were set as previously reported (Simirgiotis et al., 2016). The LC parameters were as follows: the column used was a UHPLC C18 column (Acclaim, 150 × 4.6 mm ID, 5 µm, Restek Corporation, Bellefonte, PA, USA) operated at 25 °C. The detection was set at 254, 280, 320 and 440 nm, while the PDA from 200–800 nm was recorded. The mobile phases were 1% formic aqueous solution (A) and acetonitrile with 1% formic aqueous solution (B). The gradient program time (min), (% of B) was as follows: (0.00, 5); (5.00, 5); (10.00, 30); (15.00, 30); (20.00, 70); (25.00, 70); (35.00, 5) and 12 min for column equilibration. The flow rate was set at 1.00 mL min^−1^, while the injection volume was 10 µL. The standards and extracts dissolved in methanol were maintained at 10 °C in the autosampler. The HESI II and other parameters for the Q-orbitrap instrument were optimized as previously reported [6].

### 3.2. Plant Material 

*Tetraglochin ameghinoi* was collected in the Central Andes area, San Juan province, Argentina (2700 m.a.s.l.) during the flowering period (January) and identified by Dr. L. Ariza Espinar in the Instituto Multidisciplinario de Biología Vegetal (IMBIV-CONICET-Universidad Nacional de Córdoba). A voucher specimen (BT30) has been deposited at the Museo Botánico de Córdoba (Córdoba, Argentina).

### 3.3. Extraction and Isolation 

The air-dried *T. ameghinoi* (1320 g) plant was extracted successively with petroleum ether (PE, 2 × 124 h × 3 L), dichloromethane (DCM, 2 × 124 h × 3 L), and methanol (MeOH, 2 × 124 h × 3 L) at room temperature that produced PE-E (3.2 g), DCM-E (8.1 g) and MeOH-E(43.8 g) extracts with *w*/*w* yields of 0.24%, 0.61% and 3.32%, respectively.

The PE-E (2.8 g) was chromatographed on a silica gel column (length 30 cm, internal diameter 4.5 cm) with 1.2 L of PE:EtOAc (ethyl acetate) 70:30 *v*/*v* to 0:100 *v*/*v* gradient, followed by MeOH. Fourteen fractions of 100 mL each were obtained. The fractions yielded 535 mg of ursolic acid acetate.

The MeOH-E (40 g) was re-suspended in water and partitioned with ethyl ether (Et_2_O) and ethyl acetate (EtOAc) to yield Et_2_O-E (0.5 g) and EtOAc-E (2.86 g). The remaining aqueous phase was lyophilized (35 g, Labconco 4 L, USA). The MeOH-soluble fraction from the EtOAc extract (2.8 g) was applied onto a Sephadex LH-20 column (length 50 cm, 3.5 cm i.d.; equilibrated to MeOH). Thirty fractions (50 mL each) were obtained. After thin layer chromatography (TLC) comparison (silica gel; EtOAc: MeOH, 9:1, as mobile phase; detection under UV light and after spraying *p*-anisaldehyde), the fractions with similar TLC patterns were combined (F1–F10 fractions).

The Fraction F5 (487 g) was applied to a silica gel column (length 50 cm, internal diameter 3 cm) and eluted with EtOAc:MeOH gradients. One hundred fractions of 10 mL each were obtained. After TLC comparison (silica gel, dichloromethane: methanol, 95:5 as the mobile phase; detection under UV light and spraying with *p*-anisaldehyde), the fractions with similar TLC patterns were combined.

From the previous analysis by TLC, F12–15 produced 13.5 mg of the compound 4′,6′-dihydroxy-2′-*O*-methoxy-acetophenone (peak 10, Table 1; Figure S1e, Supplementary material). The spectroscopic data are consistent with those previously reported [44,45].

The fractions 42–60 (75 mg) were found to contain a new phloracetophenone glucoside 4′,6′-dihydroxy-2′-*O*-(6′′-acetyl)-*β*-d-glucopyranosylacetophenone. 

The fractions 70–74 (96 mg) were successively chromatographed on a silica gel column (silica gel, DCM: EtOAc, 10:90 as the mobile phase; detection under UV light and spraying with p-anisaldehyde and heating), yielding 2′,6′-dihydroxy-4′-*O*-*β*-d-glucopyranosylacetophenone (40 mg) and 4′,6′-dihydroxy-2′-*O*-*β*-d-glucopyranosylacetophenone (32 mg). ^1^HNMR and ^13^CNMR data of those glycoside compounds are consistent with those previously reported [27,28,29,30,31,32,33,34,35,36,37,38,39,40,41,42,43,44,45,46].

### 3.4. Determination of Total Phenolics (TP) and Flavonoids (F) Content

The total phenolics (TP) and flavonoids (F) content of the extracts were determined by Folin–Ciocalteu and AlCl_3_ colorimetric methods, respectively [47,48]. The TP were expressed as grams of gallic acid equivalents (GAE) per 100 g of extracts (g GAE/100 g extract). F were expressed as g of quercetin equivalents (QE) per 100 g of extracts on (g QE/100 g extracts). The values from triplicates were reported as the mean ± SD.

### 3.5. Antioxidant Activity

#### 3.5.1. DPPH Scavenging Activity 

Free radical scavenger activity on DPPH free radical scavenging effects were assessed by the procedure previously described in Reference [49]. The scavenging activities were evaluated at 517 nm in a Multiskan FC microplate photometer (Thermo Scientific, Waltham, MA, USA). The analyses were performed in triplicate and values were reported as EC50 mean ± SD; being EC50, the extracts’ concentration provided 50% of radicals scavenging activity. Quercetin was used as a reference compound.

#### 3.5.2. Ferric-Reducing Antioxidant Power Assay (FRAP)

FRAP assay was performed in accordance to a previous study [50] with some modifications. Briefly, the FRAP solution was freshly prepared by mixing 10 mL of acetate buffer with a concentration of 300 mM at a pH of 3.6, 1 mL of ferric chloride hexahydrate with a concentration of 20 mM dissolved in distilled water and 1 mL of 2,4,6-tris(2-pyridyl)-*s*-triazine with a concentration of 10 mM dissolved in HCl with a concentration of 40 mM. A total of 10 µL of the sample solution was mixed with 190 µL of the FRAP solution and placed in 96-well microplates, which was performed in triplicate. The results were obtained by linear regression from a calibration plot obtained with Trolox (0–1 mmol L^−1^). All samples were analyzed in triplicate. The results were expressed as mM TE/g extract.

#### 3.5.3. Trolox Equivalent Antioxidant Activity (TEAC) Assay

The TEAC assay was performed in accordance to a previous study [51] with minor modifications. Briefly, 10 µL of the sample or Trolox standard was mixed with 200 µL of ABTS^•+^ (dissolved in PBS). The vortex was mixed for 10 s and the absorbance at 734 nm after a 4 min reaction at 30 °C was measured. The results were obtained by linear regression from a calibration plot constructed with Trolox (0–1 mmol L^−1^) and are expressed in TEAC values [52]. The TEAC value of samples is equivalent to the concentration of a Trolox solution. All samples were analyzed in triplicate. The results were expressed as mg TE/g extract.

#### 3.5.4. Lipid Peroxidation in Human Erythrocytes

The evaluation of lipid peroxidation in human erythrocytes was carried out as described by a previous study [49] with minor modifications. Human red blood cells obtained from healthy adult individuals were washed three times in cold phosphate buffered saline (PBS) by centrifugation at 3500 rpm. After washing, the cells were suspended in PBS, regulating the density to 1 mM hemoglobin in each reaction tube. The final cell suspension was incubated with different concentrations of the test compounds and dissolved in DMSO and PBS for 10 min at 37 °C. The final concentration of samples and controls in DMSO was 1%. After incubation, the cells were exposed to tert-butylhydroperoxide (1 mM) for 15 min at 37 °C under vigorous shaking. After this, the lipid peroxidation was determined indirectly by the TBARs formation. The results are expressed as a percentage of inhibition compared to the controls. Each determination was performed in quadruplicate.

### 3.6. Antibacterial Activity

The microorganisms included the Gram-positive *Staphylococcus aureus* methicillin-sensitive ATCC 29213, *Staphylococcus aureus* methicillin-resistant ATCC 43300, clinical isolates of *Staphylococcus coagulase* negative-502 and *Streptococcus pyogenes*-1 (by Laboratorio de Microbiología, Hospital Marcial Quiroga, San Juan, Argentina). Furthermore, we also used Gram-negative *Escherichia coli* ATCC 25922 and clinical isolates of *Escherichia coli* LM-2 (Laboratorio de Microbiología, Facultad de Ciencias Médicas, Universidad Nacional de Cuyo, Mendoza, Argentina).

An antibacterial susceptibility test was conducted, in which the minimal inhibitory concentration (MIC) values were determined using the broth microdilution method according to the protocols of the Clinical and Laboratory Standards Institute previously reported in a previous study [24]. The bacteria inoculum employed was 5 × 10^5^ CFU/mL. The stock solutions of extracts in the DMSO were prepared to give serial two-fold dilutions to obtain the final concentrations of 0.98–1000 µg/mL. Cefotaxime (Argentia^®^) was included in the assays as a positive control. The plates were incubated for 24 h at 37 °C. The activity was evaluated at 620 nm using a Multiskan FC instrument. The MIC values were defined as the lowest extract concentrations showing no bacterial growth after the incubation time. Tests were done in triplicate.

### 3.7. Statistical Analysis

Determinations of TP, TF, TA, DPPH, FRAP, and TEAC were performed in triplicate and the results are expressed as mean values ± SD. The results were analyzed by one-way ANOVA and significant differences between mean values were determined by Duncan’s test (*p* < 0.05). The statistical package InfoStat 26 was used for statistical analyses. Pearson’s correlation analysis was also used.

## 4. Conclusions

The identification of the main antioxidant compounds by UHPLC Orbitrap (Q-OT) and the isolation of major acetophenones derivatives in addition to the strong free radical scavenging activity of this plant supports its ethnopharmacological use to treat hepatic disorders and as a cholagogue in Andean traditional medicine to a certain extent. Further investigations are required focused to determine the potential pharmacognostical/pharmacological effects of these native plant extracts as natural sources for the preparation of phyto-pharmaceutical products.

## Figures and Tables

**Table:** (**a**) Antioxidant properties of *T. ameghinoi* extracts.

Assay	Extracts		
*Phenols Content*	DCM-E	MeOH-E	EtOAc-E
Total phenolics (mg GAE/g extract)	16.62 ± 3.18 ^a^	107.15 ± 7.78 ^b^	45.43 ± 7.51 ^c^
Flavonoids (mg QE/g extract)	nd	19.93 ± 1.61	nd
*Antioxidant*			
DPPH (IC_50_ in µg/mL)	nd	17.70	45.01
FRAP (mM TE/g extract)	67.69 ± 8.27 ^a^	381.43 ± 22.38 ^b^	288.32 ± 43.24 ^c^
TEAC (mg TE/g extract)	85.42 ± 12.37 ^a^	387.76 ± 91.93 ^b^	154.73 ± 12.42 ^c^
Percentage LP (at 100 µg/mL)	12.28 + 1.29	93.23 + 6.77	

Different letters indicate significant difference among extracts, Tukey (*p* < 0.0001); nd: not detected.

**Table:** (**b**) Antimicrobial properties of *T. ameghinoi* extracts.

Assay	Extracts			
*Antibacterial*	DCM-E	MeOH-E	EtOAc-E	Cefotaxime
CLSI (MICs in µg/mL)				
*Staphylococcus aureus* methicillin-sensitive ATCC 29213	750	750	750	0.5
*Staphylococcus aureus*, methicillin-resistant ATCC 43300	750	750	750	0.5
*Staphylococcus aureus*, coagulase negative-502	750	750	>1000	0.5
*Streptococcus pyogenes*-1	>1000	>1000	>1000	0.25
*Escherichia coli* ATCC 25922	>1000	>1000	>1000	1.9
*Escherichia coli-LM_2_*	>1000	>1000	>1000	1.5

**Table 2:** The UHPLC-DAD-OT-HR-MS identification of metabolites in *T. ameghinoi* MeOH-E.

Peak	Tentative Identification	RT (min)	UV Max (nm)	[M − H]^−^	Theoretical Mass (*m*/*z*)	Measured Mass (*m*/*z*)	Accuracy (δppm)	MS^n^ ions
1	Dihexose	1.23	-	[C_12_H_22_O_11_ + Cl]^−^	377.08506	377.08365	−3.73	341.00042
2	Dihexose	1.50	-	[C_12_H_22_O_11_ + Cl]^−^	377.08506	377.08368	−3.65	
3	Unknown	1.82	-	C_13_H_23_O_13_N_3_^−^	429.12259	429.12259	−1.10	
4	4′,6′-dihydroxy-2′-*O*-*β*-d-glucopyranosyl-acetophenone *	8.71	236, 285	C_14_H_17_O_9_	329.08781	329.08612	−1.10	167.03342 (C_8_H_7_O_4_^−^)
5	2′,6′-dihydroxy-4′-*O*-*β*-d-glucopyranosyl-acetophenone *	8.73	236, 285	C_14_H_17_O_9_^−^	329.08781	329.08618	−1.12	167.03345 (C_8_H_7_O_4_^−^)
6	4-hydroxy-2′-*O*-arabinopyranosyl-6-*O*-galloyl reduced acetophenone derivative	9.07	282	C_20_H_21_O_12_^−^	453.10148	453.10156	−3.6	301.03381 C_13_H_17_O_8_^−^), 169.01360
7	Ellagic acid hexoside	9.68	253, 367	C_20_H_15_O_13_^−^	463.05181	463.04950	−4.98	299.07567
8	Unknown	9.90	-	C_21_H_5_O_7_N^−^	383.00610	383.00610	5.46	245.04752
9	Dimethyl gallate hexoside derivative	10.11	280	C_21_H_23_O_11_^−^	451.12234	451.12234	0	
10	4′,6′-dihydroxy-2′-*O*-methoxy acetophenone *	10.32	247, 280	C_9_H_9_O_4_^−^	181.04903	181.05063	8.87	167.03337 (C_8_H_7_O_4_^−^)
11	Quercetin-3-*O*-(6’-*O*-galloyl)-hexose	10.44	254, 280, 354	C_28_H_23_O_16_^−^	615.09916	615.09518	−5.8	463.08565
12	4-hydroxy-2′-*O*-*β*-d-glucopyranosyl-6-*O*-galloylacetophenone	10.54	250, 281	C_21_H_21_O_13_^−^	481.09876	481.09613	5.46	
13	Ellagic acid *	10.76	255, 366	C_14_H_5_O_8_^−^	300.99750	300.99899	4.9	151.00217
14	Ellagic acid methyl ether hexoside	10.89	255, 366	C_21_H_17_O_13_^−^	477.06746	477.06494	−5.2	
15	4′,6′-dihydroxy-2′-*O*-(6′′acetyl)-*β*-d-glucopyranosylacetophenone	11.13	234, 285	C_16_H_19_O_10_^−^	371.09837	371.09653	−4.9	167.03355 (C_8_H_7_O_4_^−^)
16	Quercetin-3-*O*-pentoside	11.24	254, 351	C_20_H_17_O_11_^−^	433.07763	433.07763	−4.7	
17	Methyl ellagic acid-*O*-phosphate	11.43	285	C_15_H_8_O_11_P^−^	394.98097	394.96957	−29	315.01288
18	Methyl ellagic acid-*O*-phosphate	11.60	285	C_15_H_8_O_11_P^−^	394.98097	394.96948	−28.8	315.01285, 299.98950
19	Ellagic Acid methylester	11.78	254, 365	C_15_H_7_O_8_^−^	315.01464	315.01306	−5.01	299.98941
20	Ellagic acid methylester	12.04	254, 365	C_15_H_7_O_8_^−^	315.01464	315.01309	−4.9	299.98929
21	Maddecasic acid	12.29		C_30_H_47_O_6_^−^	503.33493	03.33493	0.25	
22	Rhamnazin 3-*O*-rhamnose (Isorhamnetin-7-*O*-methyl ether, 3-*O*-rhamnose)	12.76	254, 354	C_23_H_23_O_11_^−^	475.12459	475.12210	−5.24	
23	Kaempferol 3-*O*-rutinoside	13.06	265, 313	C_2_7H_29_O_15_^−^	593.15119	593.12695	−2.9	285.03824
24	Quercetin *	13.52	255, 355	C_15_H_9_O_7_^−^	301.03538	301.03384	−5.11	
25	Syringetin-3-*O*-hexoside	14.24	254, 354	C_23_H_23_O_13_^−^	507.11441	507.11154	−2.86	
26	Unknown flavonol derivative	19.76	287	C_29_H_53_O_15_N^−^	655.34509	655.34509	−10	301.14301
27	Bartogenic acid	20.29	-	C_30_H_45_O_7_^−^	517.31408	517.31421	0.25	
28	Gingerol	21.04	255, 355	C_17_H_25_O_4_^−^	293.17583	293.17776	−6.58	
29	Unknown	21.32	287	C_28_H_17_ON_2_^−^	397.13300	-	-	

* Identified by spiking experiments with authentic standards.

## Supplementary Materials

The following are available online.
