# Supplementary material for: UHPLC-MS Metabolome Fingerprinting: The Isolation of Main Compounds and Antioxidant Activity of the Andean Species Tetraglochin ameghinoi (Speg.) Speg
Source: Molecules. 2018 Mar 29;23(4):793. doi: 10.3390/molecules23040793 (PMC6017344; doi:10.3390/molecules23040793)

**Supplementary material: UHPLC-MS Metabolome Fingerprinting: The Isolation of Main Compounds and Antioxidant Activity of the Andean Species *Tetraglochin ameghinoi* (Speg.) Speg.**

**Lorena Luna, Mario J. Simirgiotis, Beatriz Lima, Jorge Bórquez, Gabriela E. Feresin and Alejandro Tapia**

**Figure S1:** Full HR MS spectra and proposed structures of a) peak 4, b) peak 6, c) peak 7, d) peak 9, e) peak 10, f) peak 15, g) peak 12, h) peak 13, i) peak 14, j) peak 15, k) peak 16, l) peak 17, m) peak 19, n) peak 22, o) peak 23, p) peak 25, q) peak 17 and r) peak 28.***
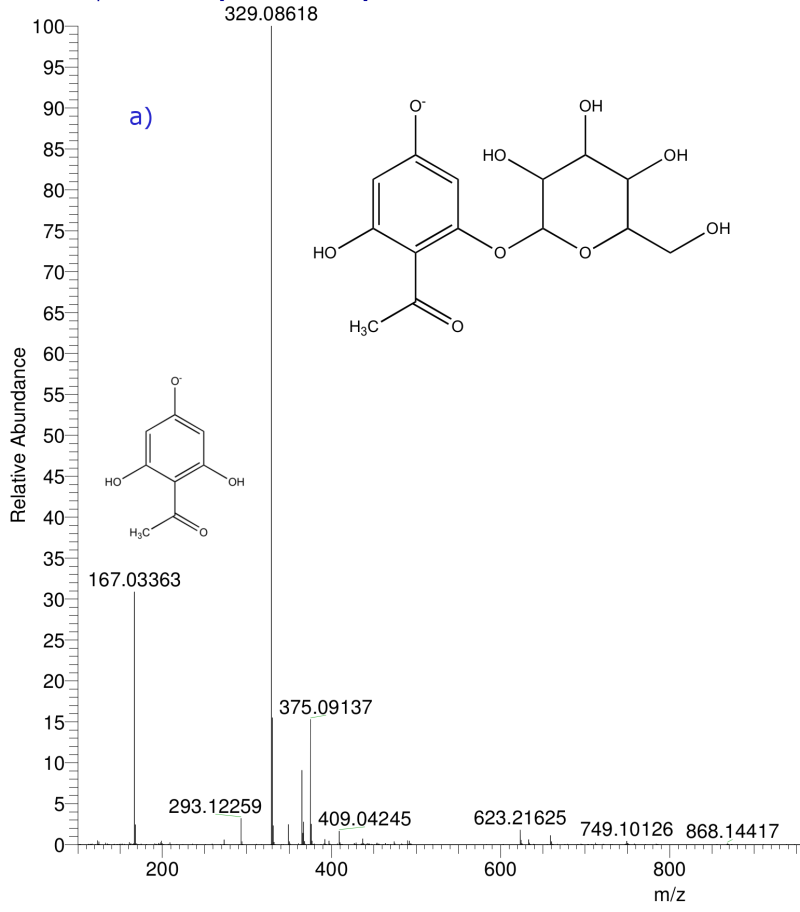
***


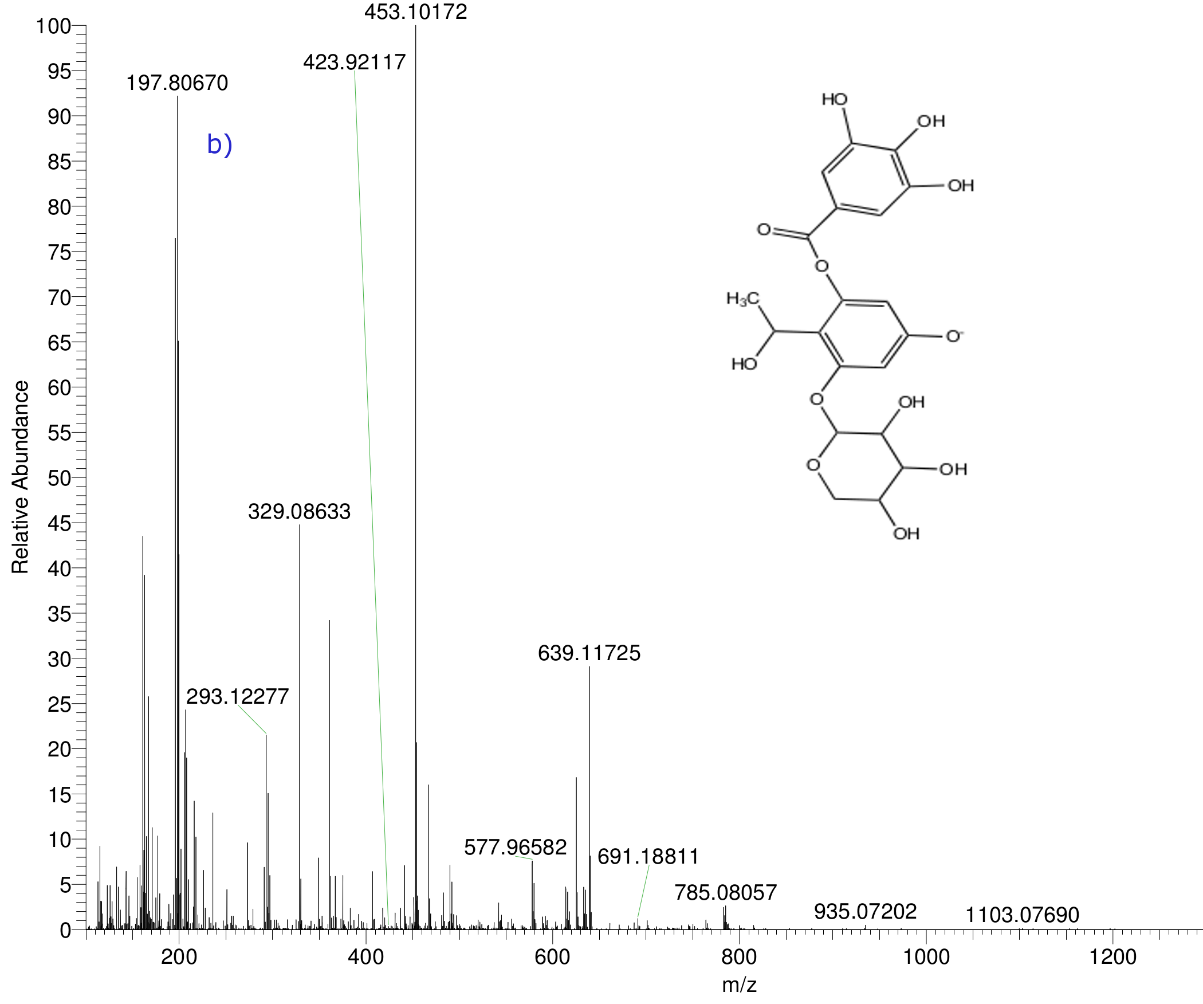


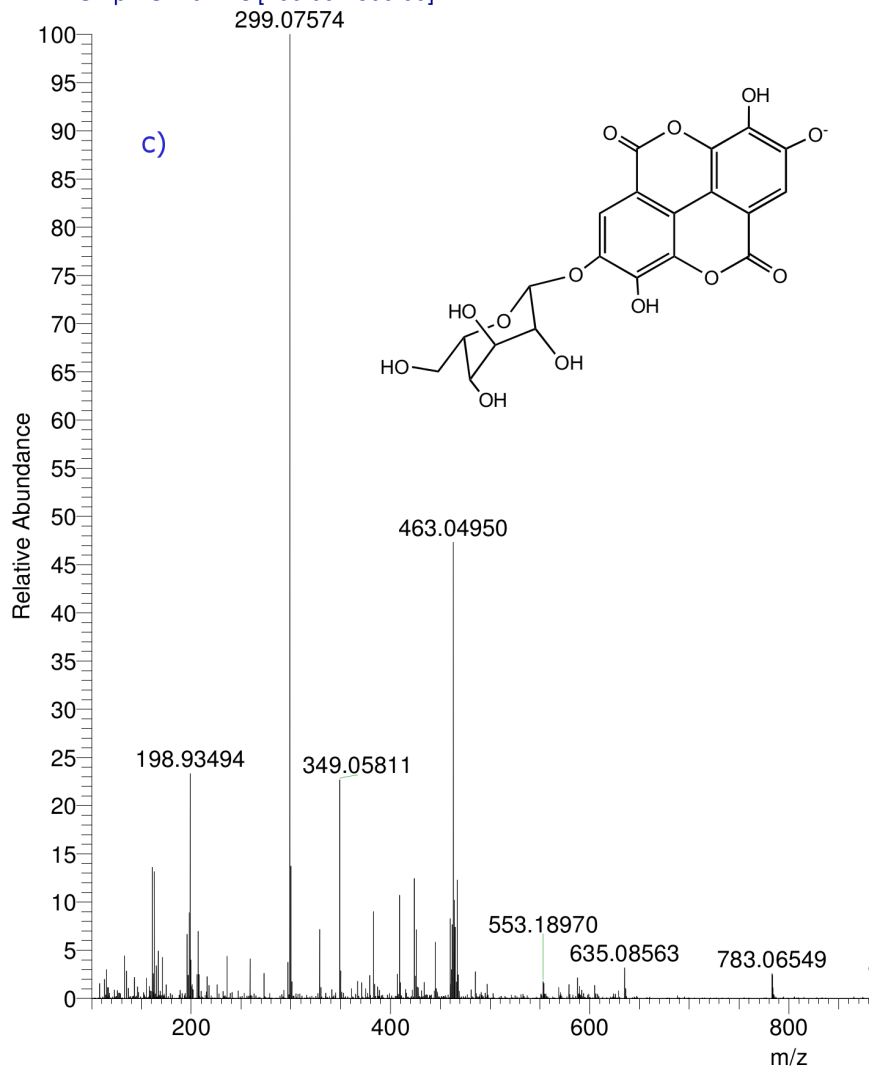

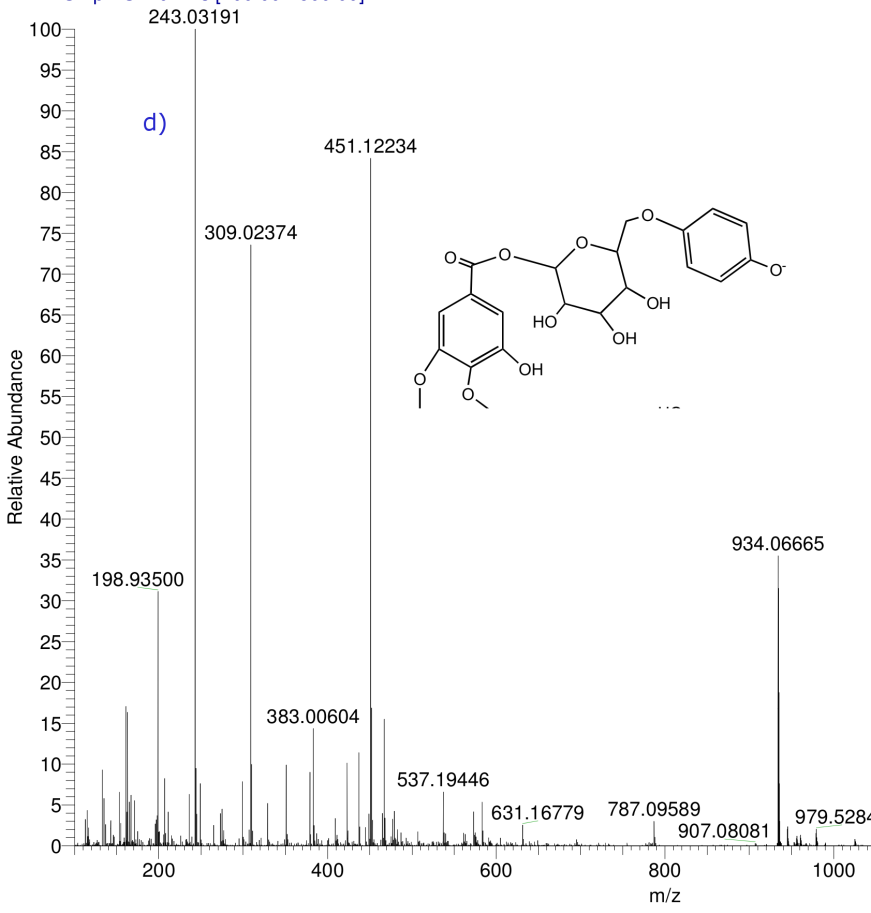

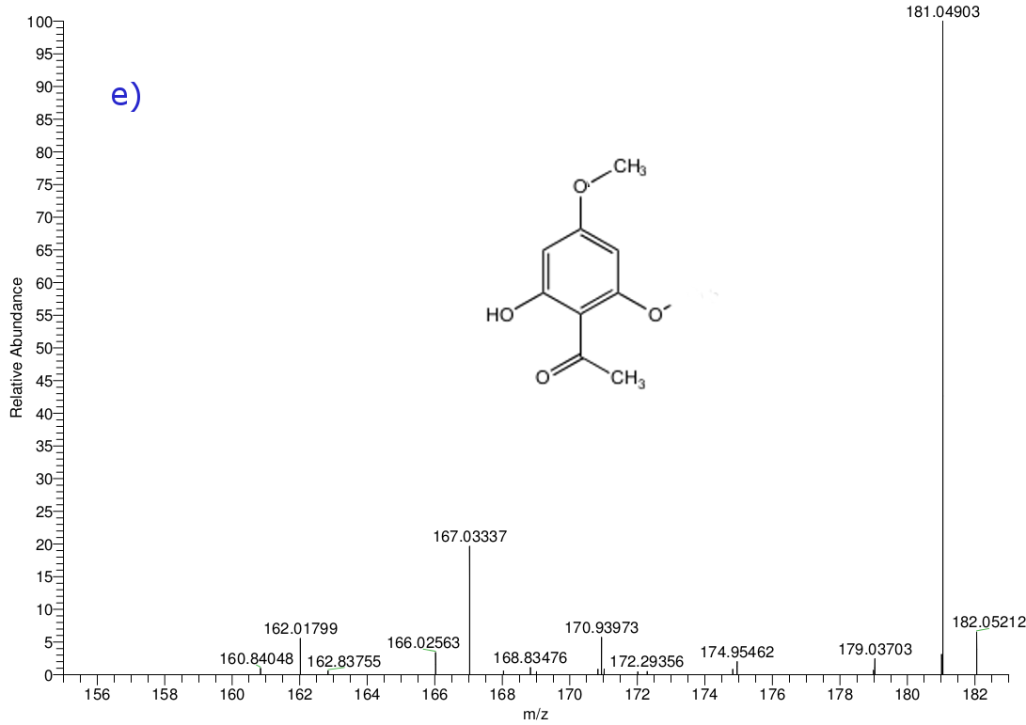


0000000000000000000000000000000000000000000000000000000000000000
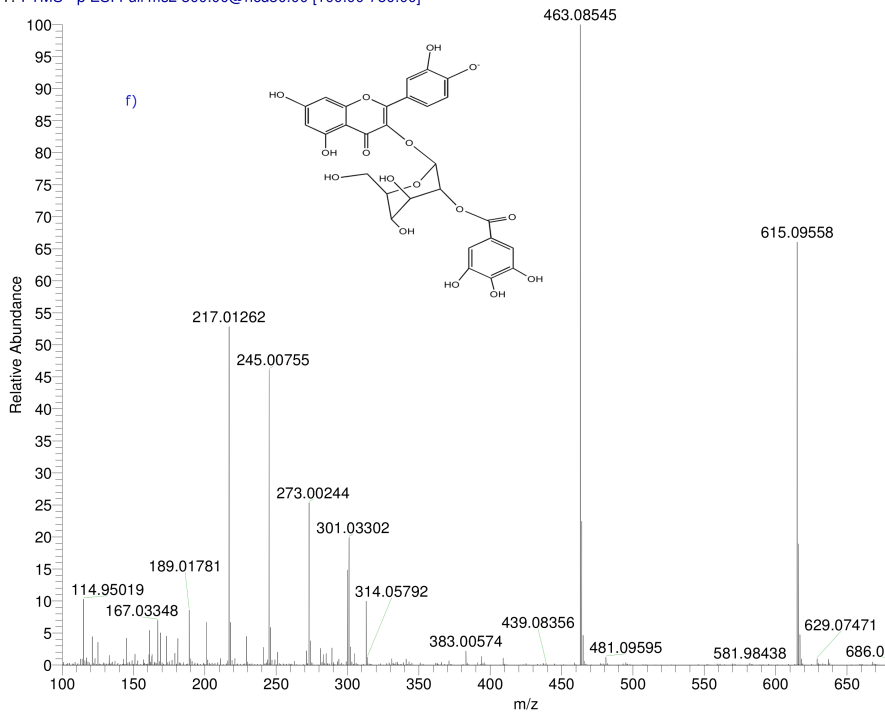


***
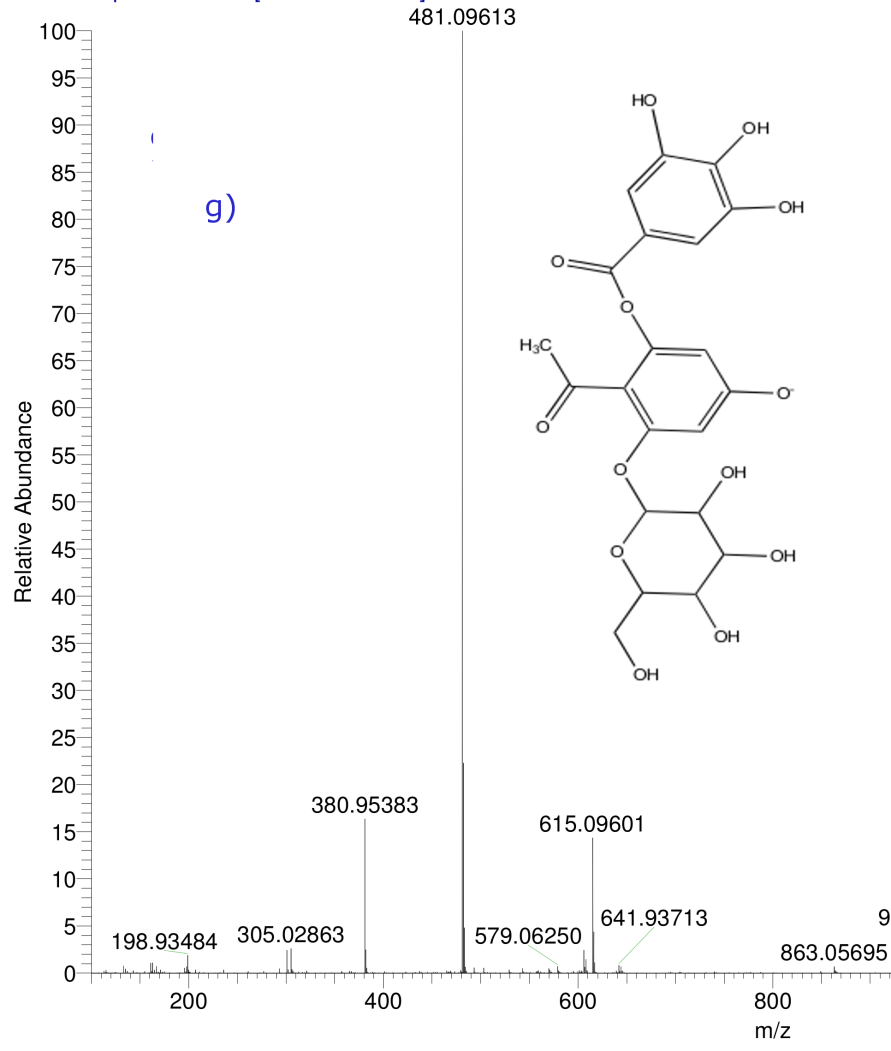

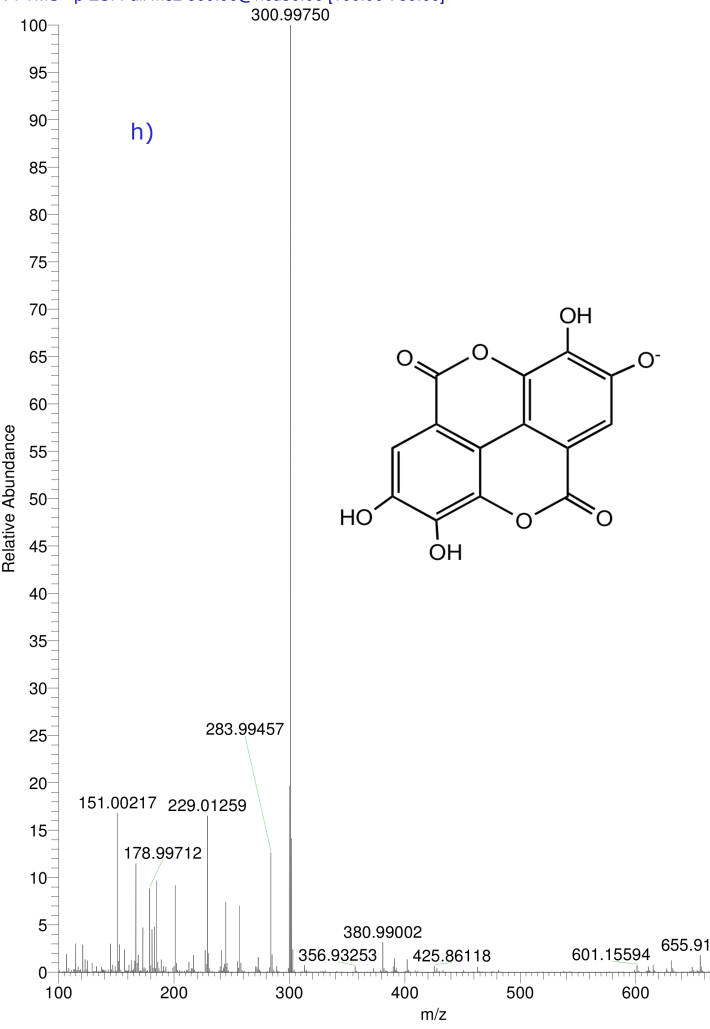
***
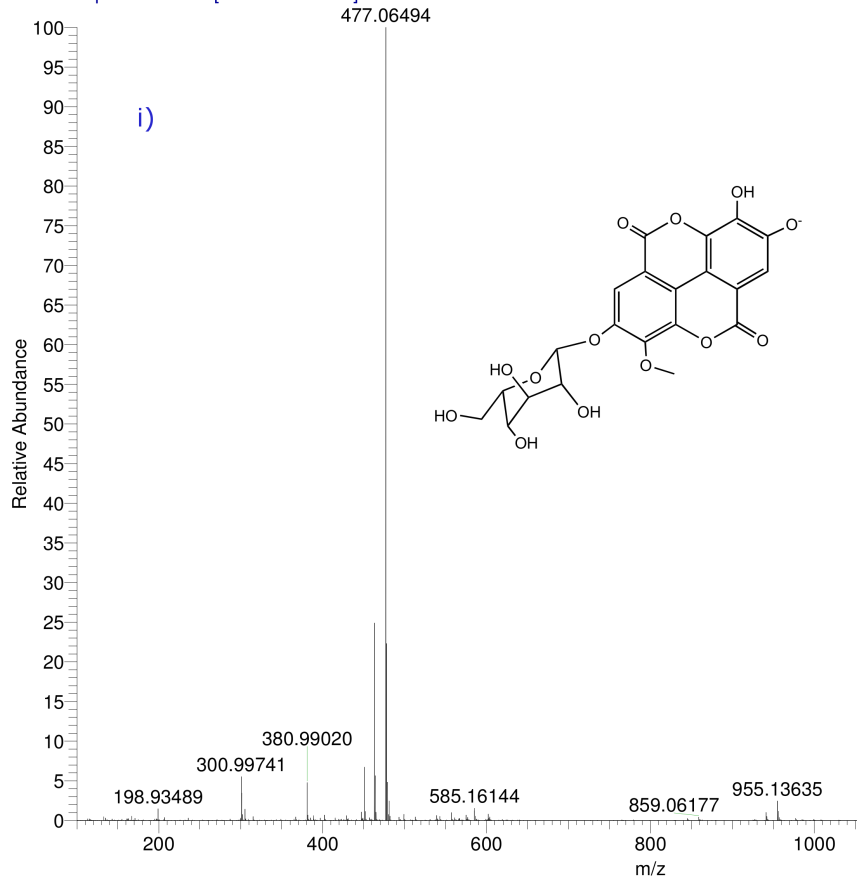

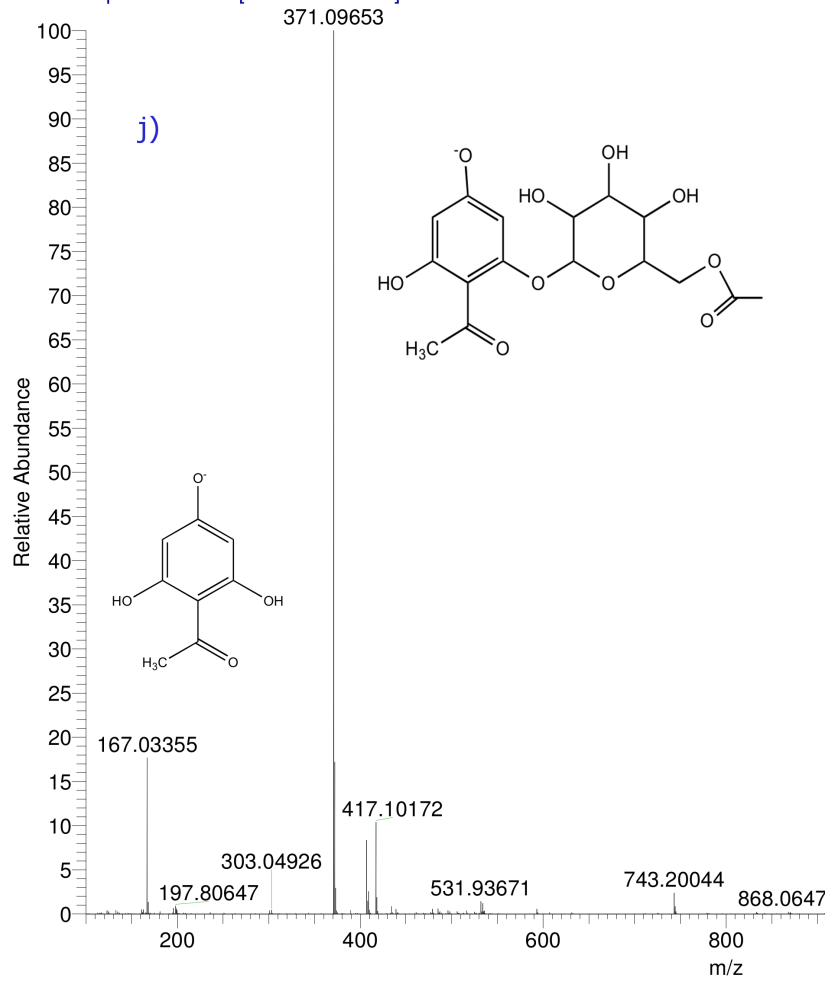

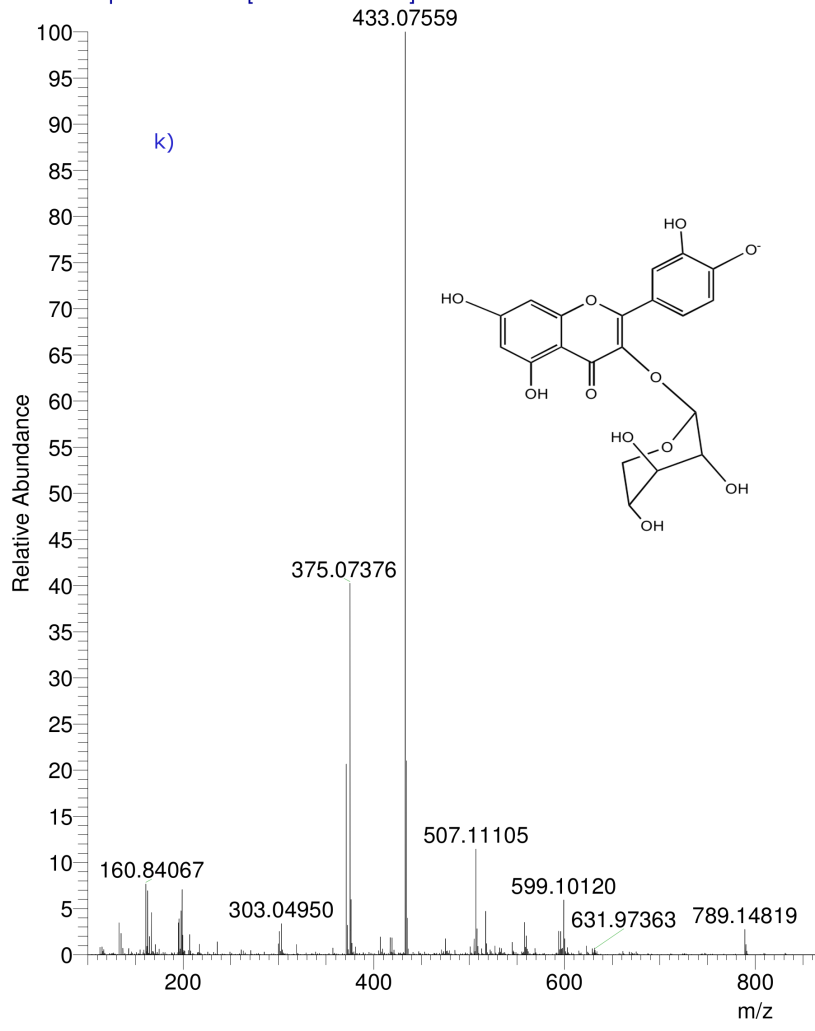

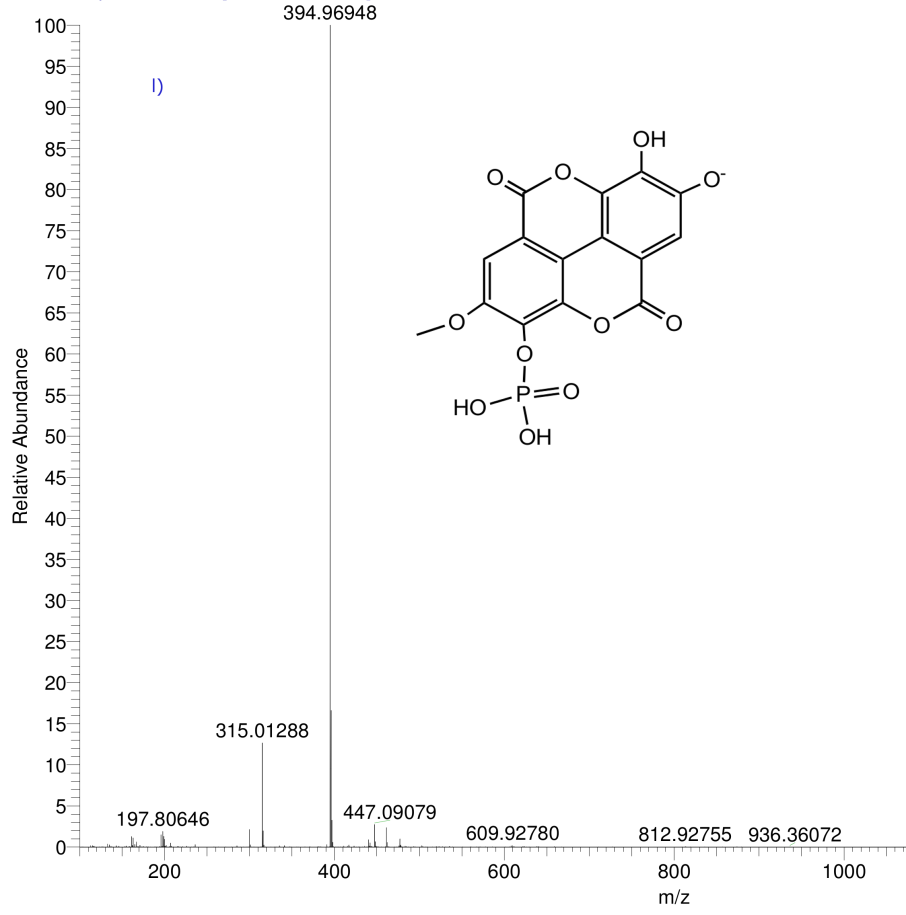

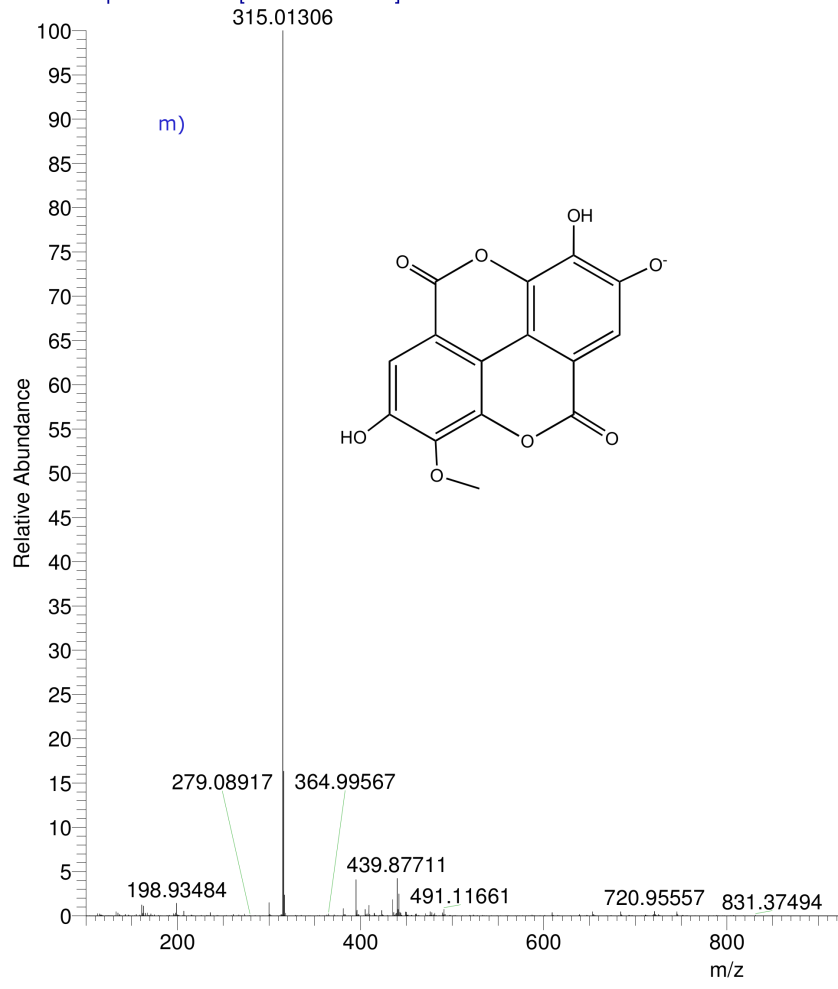

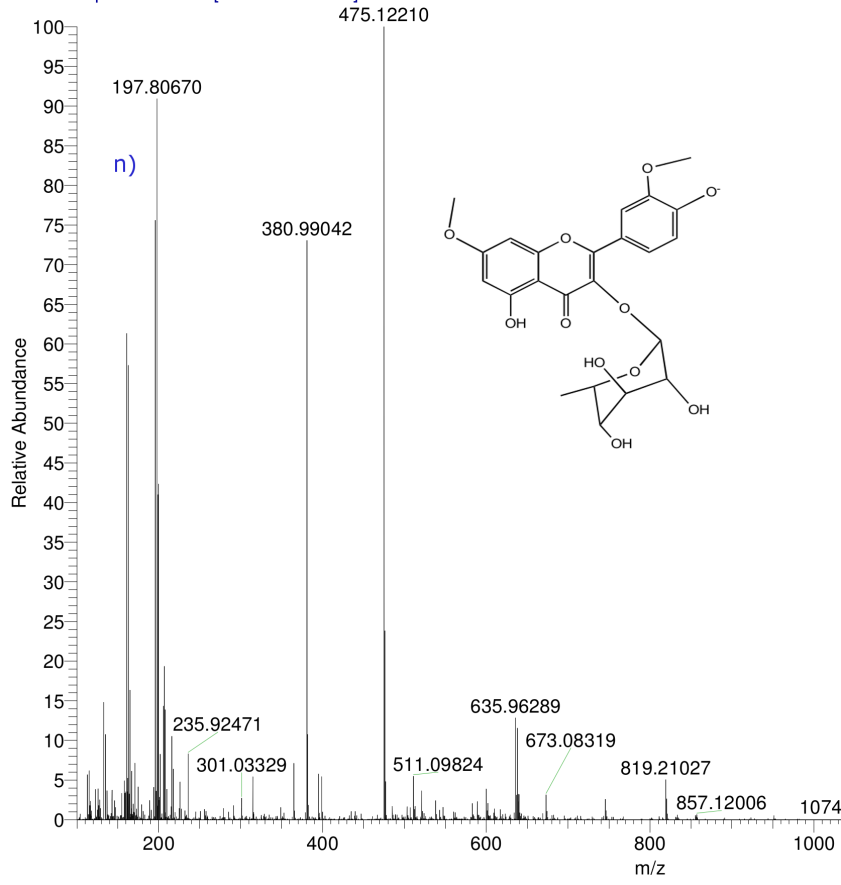

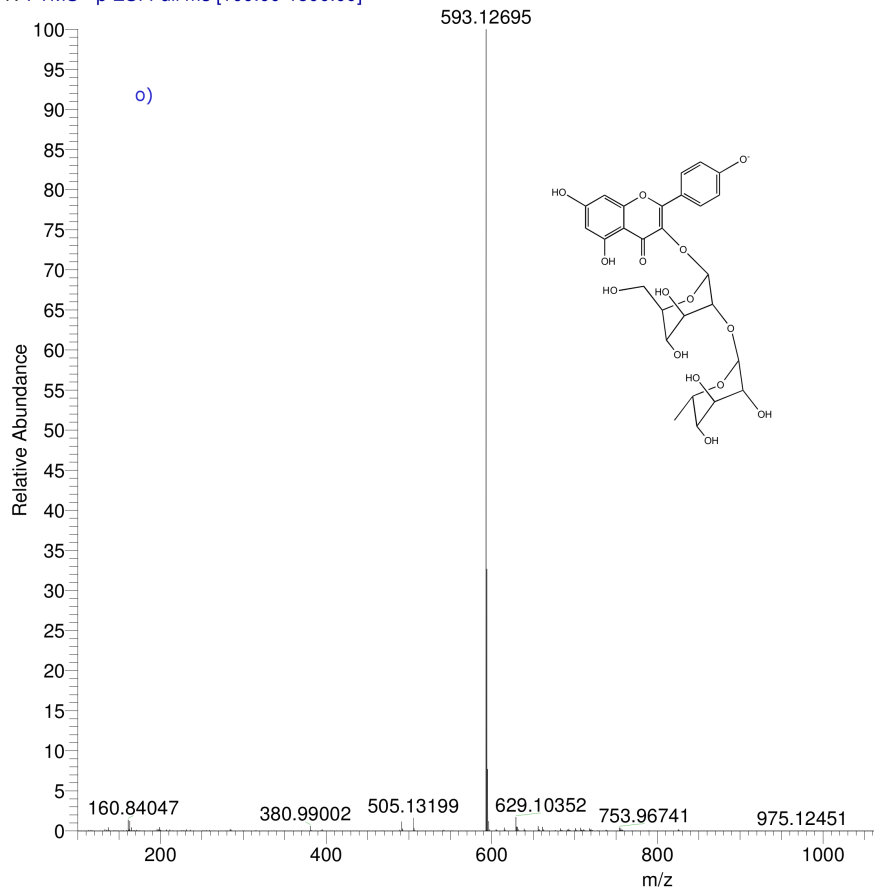


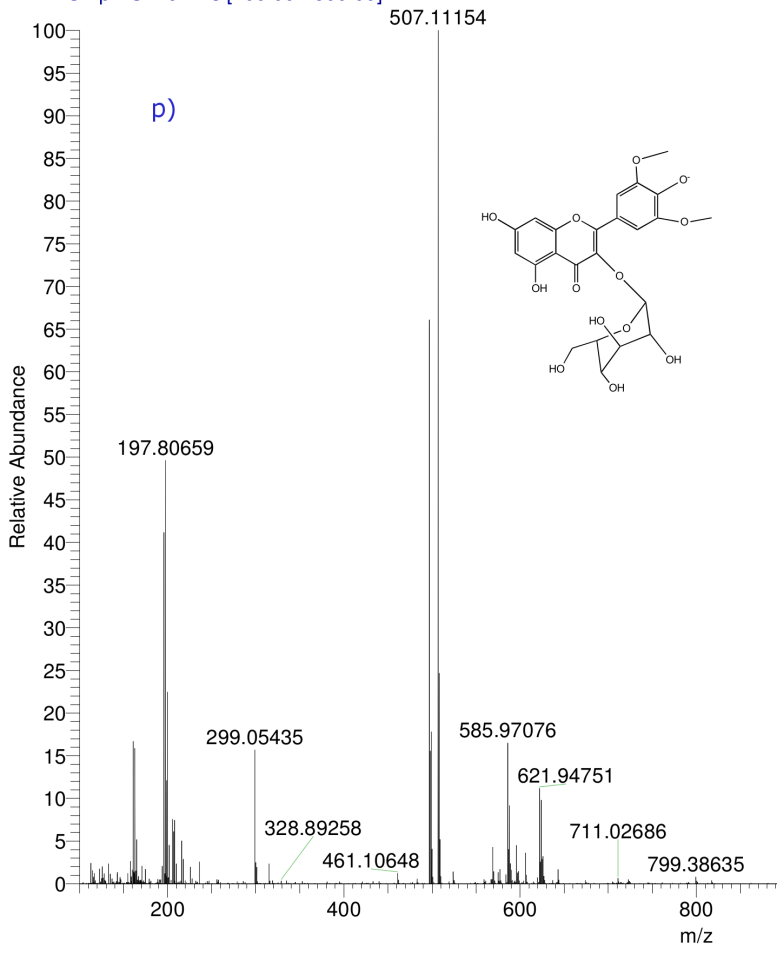

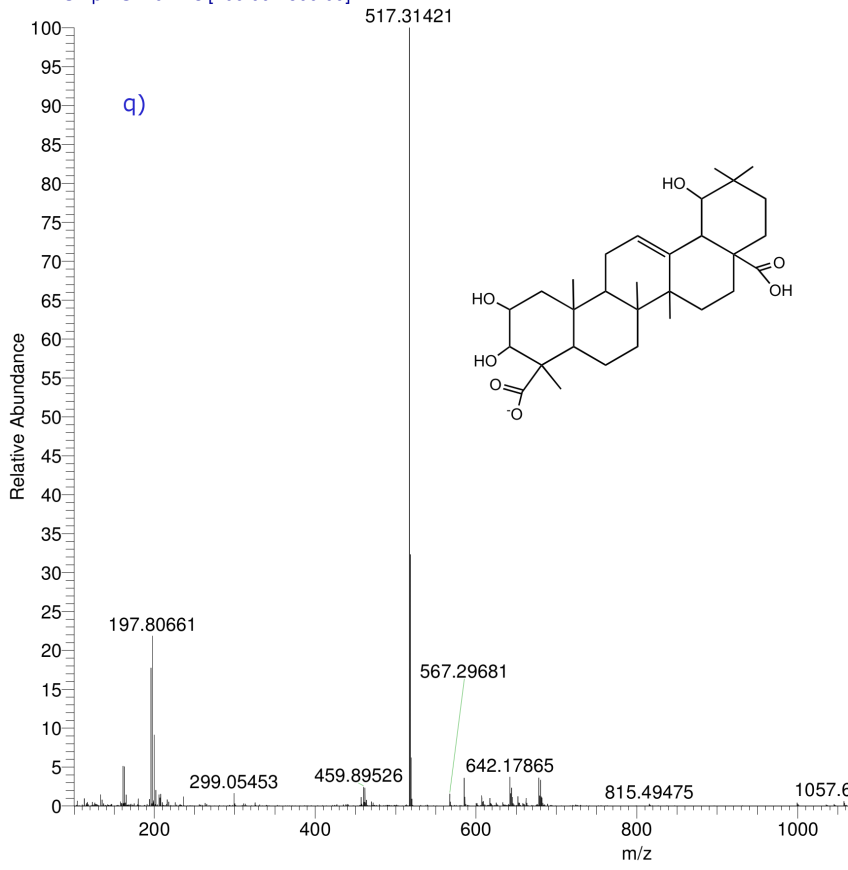

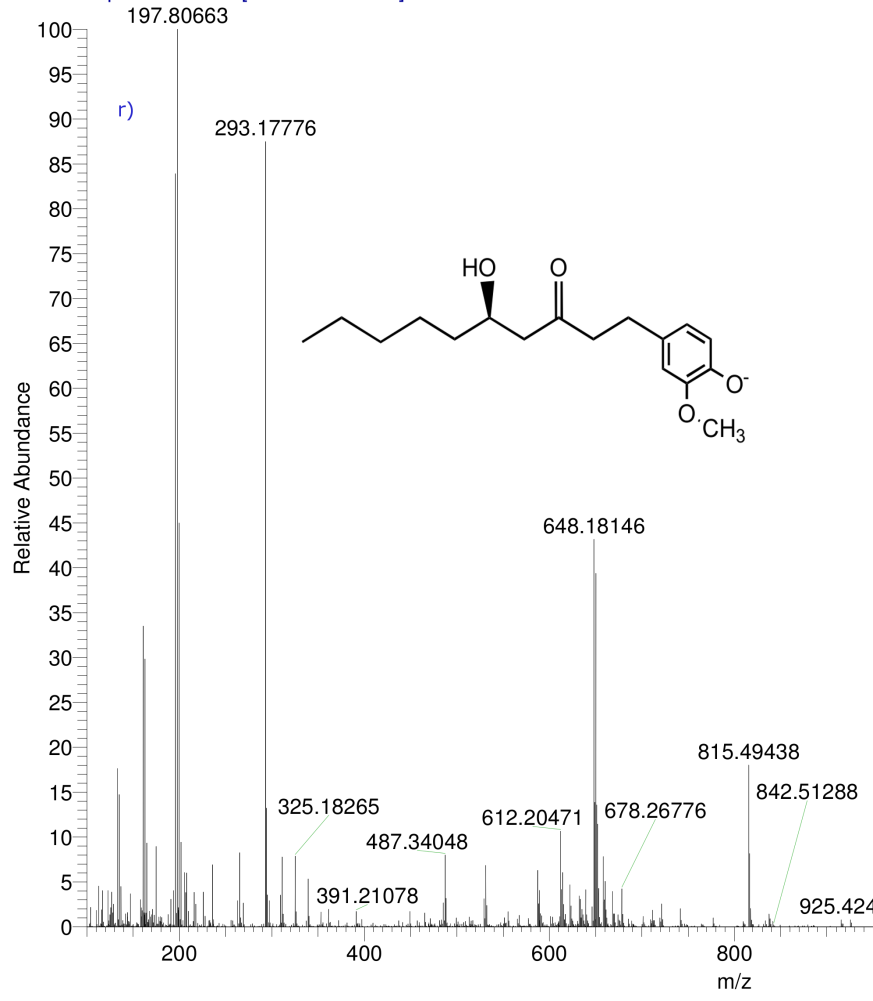


^1^H NMR (400MHz, MeOD) spectrum of the new compound 4´,6´-dihydroxy-2´-O-(6´´-acetyl)-β-D-glucopyranosylacetophenone

^13^C NMR (125MHz, MeOD) spectrum of the new compound 4´,6´-dihydroxy-2´-O-(6´´-acetyl)-β-D-glucopyranosylacetophenone


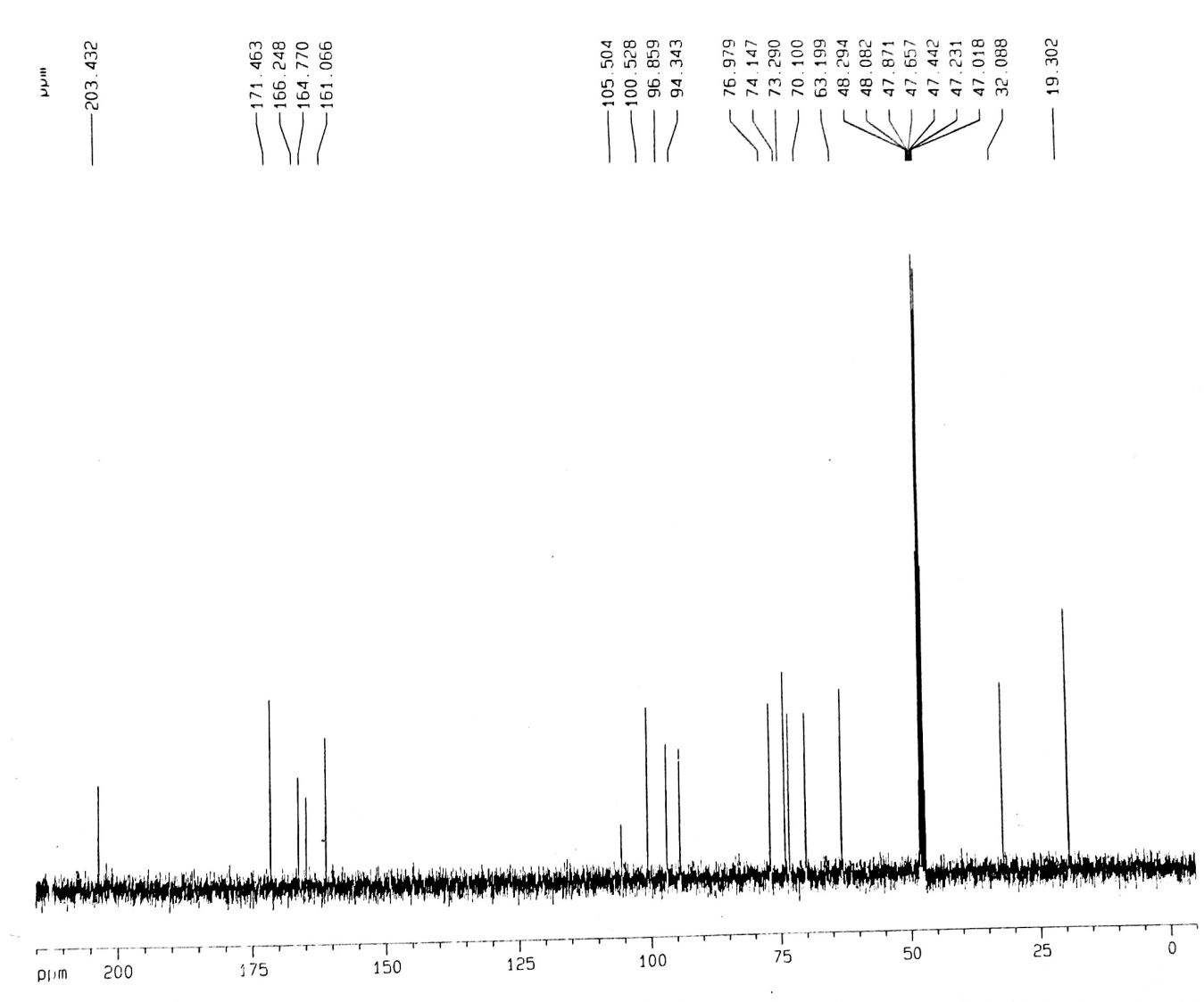


DEPT135 (125MHz, MeOD) spectrum of thenew compound 4´,6´-dihydroxy-2´-O-(6´´-acetyl)-β-D-glucopyranosylacetophenone


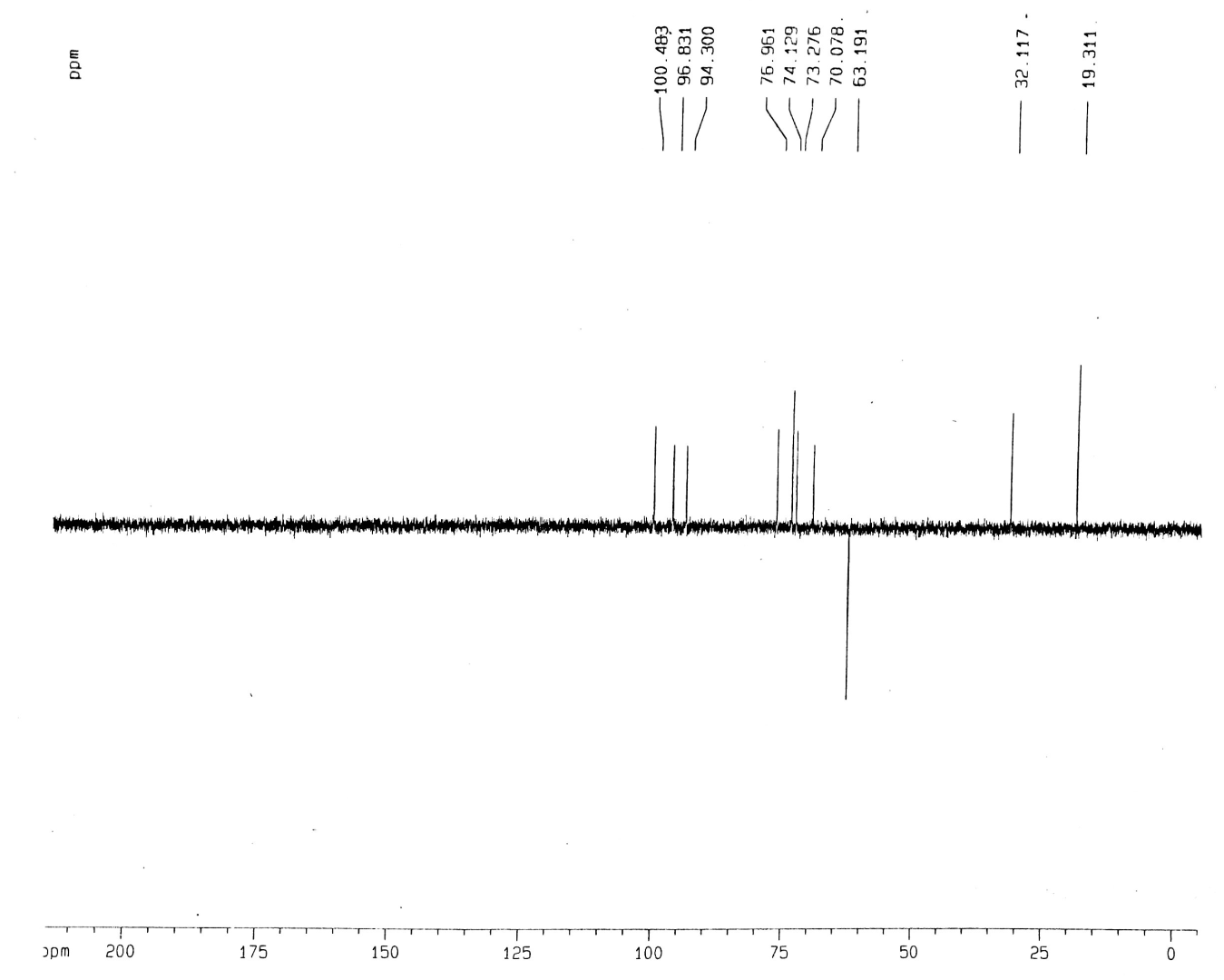


COSY (400MHz, MeOD) spectrum of the new compound 4´,6´-dihydroxy-2´-O-(6´´-acetyl)-β-D-glucopyranosylacetophenone


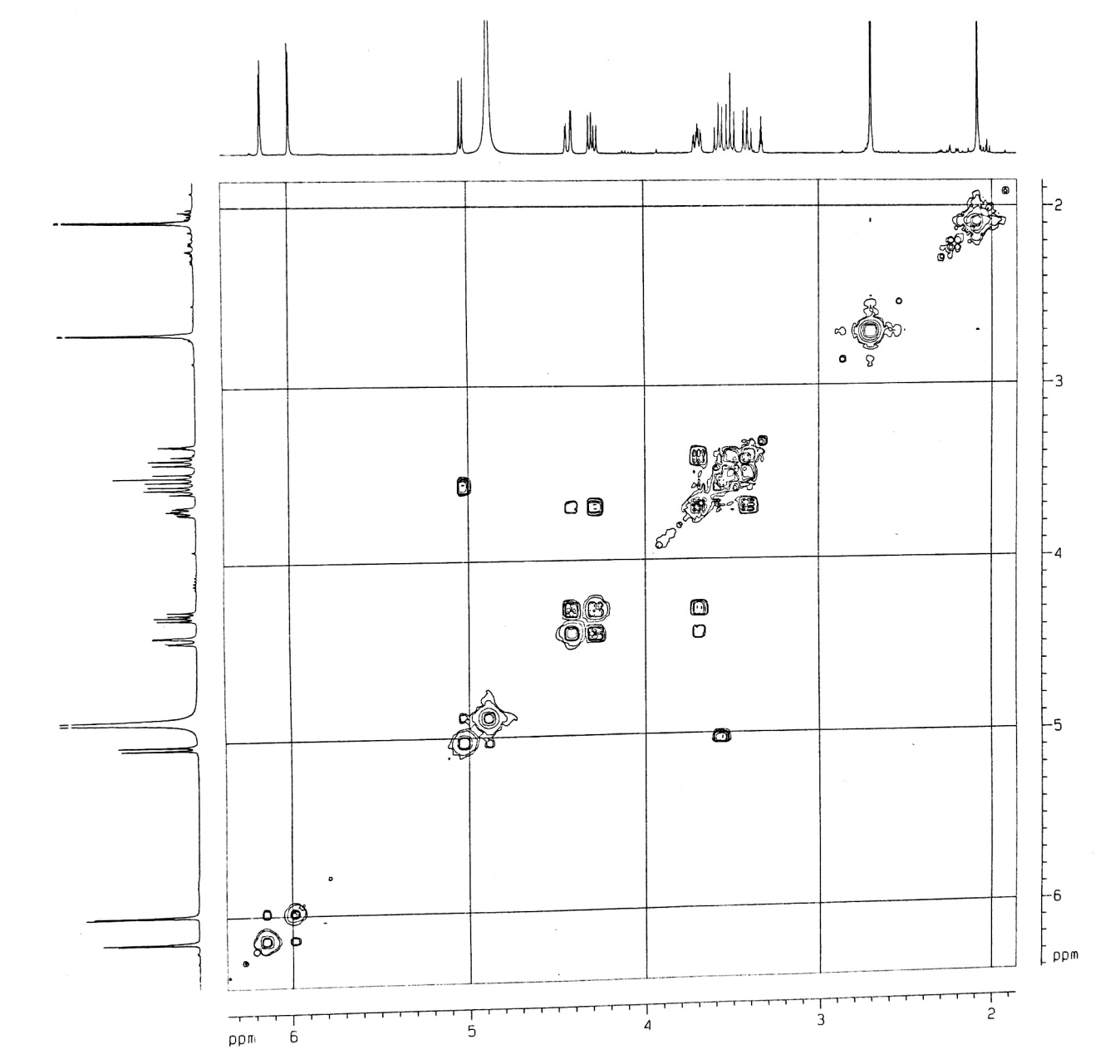


HSQC (400MHz, MeOD) spectrum of the new compound 4´,6´-dihydroxy-2´-O-(6´´-acetyl)-β-D-glucopyranosylacetophenone


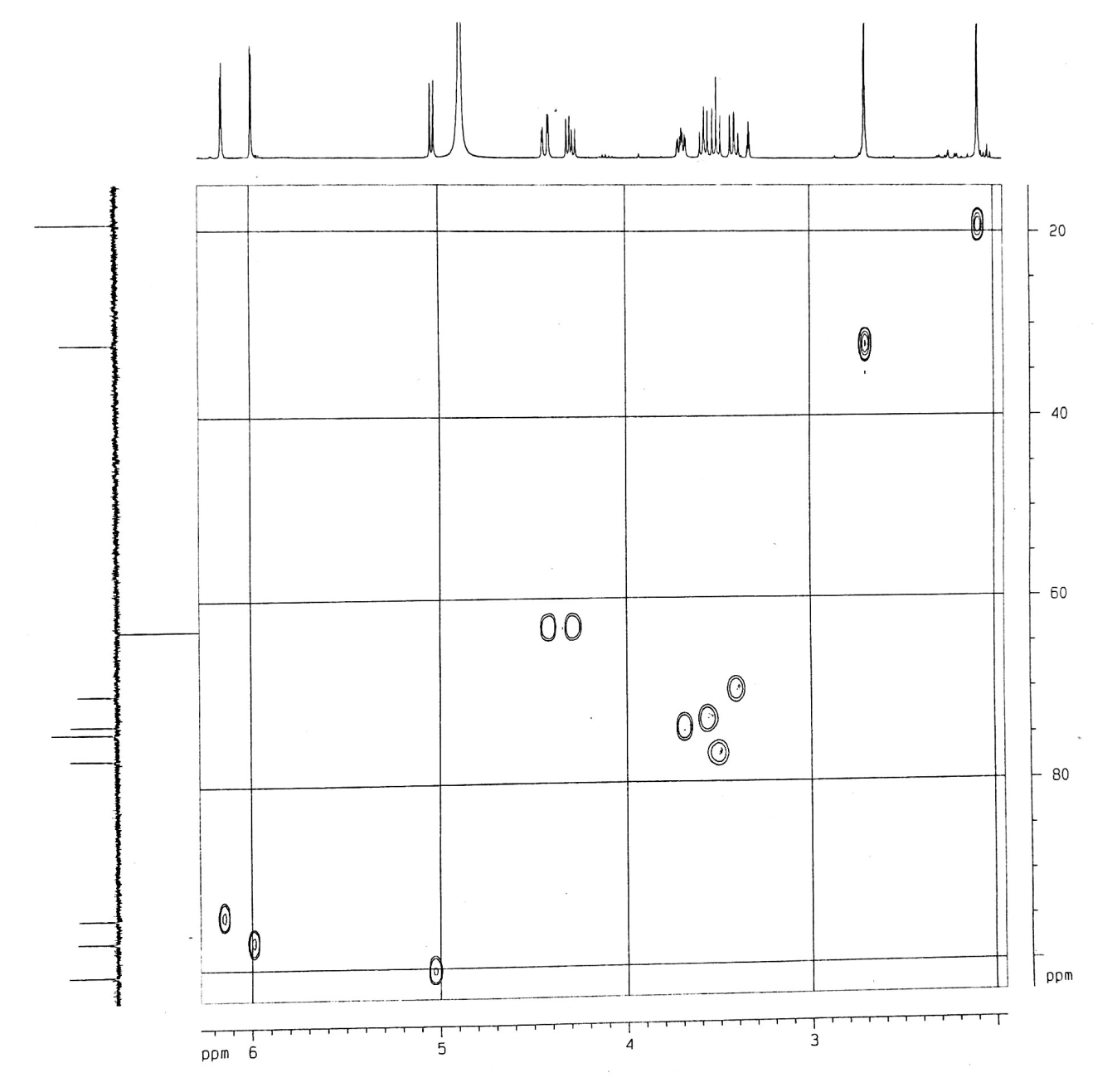


HMBC (400MHz, MeOD) spectrum of the new compound 4´,6´-dihydroxy-2´-O-(6´´-acetyl)-β-D-glucopyranosylacetophenone


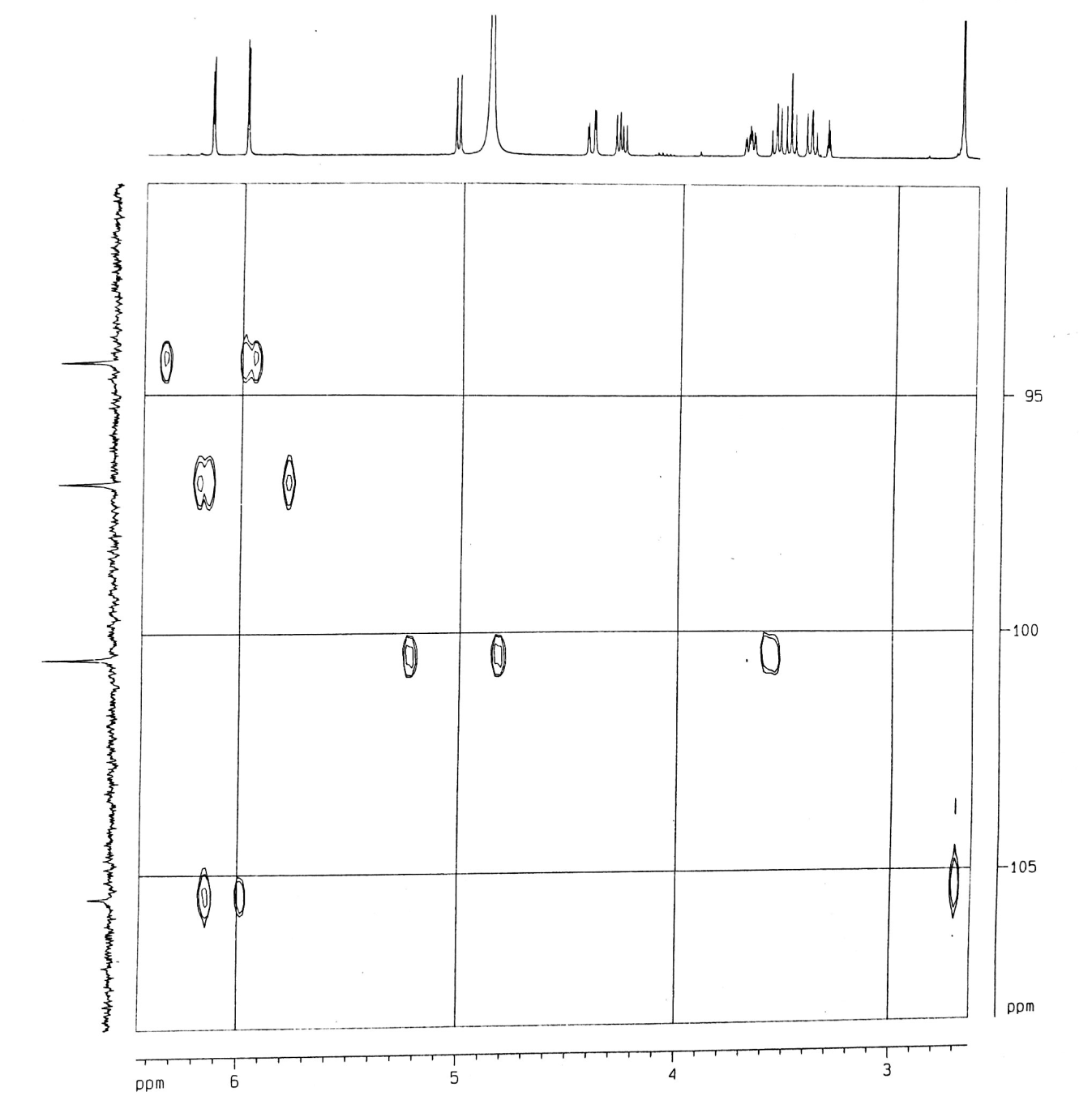

Supplement: Supplementary file 1 [file molecules-23-00793-s001.zip › Supplementary material Luna et al.,with corrections.docx]
